# Radon Exposure: A Comprehensive Review of the Health Risks and Therapeutic Potentials

**DOI:** 10.3390/toxics14100912

**Published:** 2026-10-15

**Authors:** Fengdi Jiang, Yifan Liu, Xiaotao Xie, Mingyu Wang, Jian Tong, Bengui Ye, Jihua Nie, Shuyu Zhang

**Affiliations:** 1 Laboratory of Radiation Medicine, West China School of Basic Medical Sciences & Forensic Medicine, Sichuan University, Chengdu 610041, China; 2 West China School of Public Health, Sichuan University, Chengdu 610041, China; 3 Jiangsu Key Laboratory of Preventive and Translational Medicine for Geriatric Diseases, Department of Toxicology, School of Public Health, Medical College of Soochow University, Suzhou 215123, China; 4 Medical College of Tibet University, Lhasa 850000, China; 5 Medical College of Key Laboratory of Drug-Targeting and Drug Delivery System of the Education Ministry, Sichuan Engineering Laboratory for Plant-Sourced Drug, and Sichuan Research Center for Drug Precision Industrial Technology, West China School of Pharmacy, Sichuan University, Chengdu 610041, China; 6 School of Medicine, Southwest University of Science and Technology, Mianyang 621010, China

**Keywords:** radon (Rn), radon therapy, lung cancer, DNA damage, oxidative stress

## Abstract

Radon, a naturally occurring radioactive gas, is classified as a Group 1 carcinogen by the World Health Organization (WHO) and remains a significant environmental health hazard. Radon exposure is the second leading cause of lung cancer worldwide, and lung cancer remains the only health outcome firmly established to be caused by radon exposure. Possible associations with chronic obstructive pulmonary disease, hematological disorders, skin diseases, and other conditions remain under investigation. This review explores the broad spectrum of health risks associated with radon exposure, including its biological effects at histopathological, cellular, and molecular levels, with a focus on unique radiation effects such as the bystander effect and inverse dose-rate effect. Despite its harmful effects, radon has also been investigated for potential therapeutic effects under controlled exposure conditions, although the strength of clinical evidence varies across indications. Known as radon therapy, it has been investigated primarily for rheumatic and musculoskeletal disorders, while evidence for neurological disorders and cancer-related applications remains largely experimental or limited. The biological mechanisms of radon therapy, including its regulation of oxidative stress, immune modulation, and redox balance, are discussed. The effects of radon on human health, whether harmful or therapeutic, are highly dependent on factors such as dose, exposure duration, and individual susceptibility. This review concludes by highlighting key areas for future research to refine our understanding of both the risks and therapeutic applications of radon.

## 1. Introduction

Radon, a naturally occurring radioactive noble gas, is a significant environmental factor influencing human health. It is colorless, odorless, and tasteless, primarily arising from the radioactive decay of uranium and thorium in soil, rock, and water [1]. Radon can accumulate in enclosed spaces, such as homes and underground workplaces, leading to chronic exposure [2]. Recent analyses have reported increases in measured indoor radon concentrations in some regions. In China, a secondary analysis of residential surveys conducted between 1980 and 2019 estimated an average increase of approximately 0.80 Bq/m^3^ per year across 28 cities [3]. However, this temporal trend should be interpreted cautiously and should not be assumed to represent a proportional increase in population exposure, as differences in survey conditions and measurement approaches across decades may influence comparisons. Due to its radioactive properties, radon exposure has been widely studied for its potential health effects, including its role in carcinogenesis and other diseases [4,5,6].

Initially, radon was primarily recognized for its harmful effects. As a well-established carcinogen, radon is strongly associated with lung cancer, as acknowledged by the WHO and the International Agency for Research on Cancer (IARC) [7,8]. Inhalation of radon and its decay products results in α-particle radiation exposure, leading to DNA damage, genomic instability, and inflammatory responses, which contribute to respiratory and systemic diseases [9,10,11]. Furthermore, radon exposure has been linked to hematological, skin, and other organ-specific disorders, raising concerns about its widespread environmental presence [12,13]. However, radon has also been investigated for potential therapeutic applications under controlled exposure conditions. Low-dose radon exposure has been reported to modulate inflammatory, immune, and oxidative stress-related responses, which have been proposed as possible mechanisms underlying therapeutic effects in selected chronic inflammatory and degenerative conditions [14,15,16]. Radon therapy has been used particularly for rheumatic disorders and has also been explored for other indications, although the strength of clinical evidence varies considerably and its underlying biological mechanisms remain under investigation [14,17].

This review aims to provide a comprehensive overview of the role of radon in diseases and therapeutic methods by examining its sources, exposure pathways, detection methods, and emphasizing the biological mechanisms underlying its pathological effects. Additionally, this review explores the potential therapeutic applications of radon, highlighting the potential mechanisms underlying biological responses to low-dose exposure. By integrating current knowledge on both the detrimental and therapeutic aspects of radon exposure, this review seeks to contribute to a more thorough and in-depth understanding of its impact on human health.

## 2. Search Strategy

A comprehensive literature search was conducted using the Web of Science Core Collection, PubMed, Scopus, and ScienceDirect to identify publications relevant to radon exposure, health effects, biological mechanisms, and potential therapeutic applications. The literature included in this review covered the period from 1981 to 2026.

The main search terms included “radon”, “radon exposure”, “residential radon”, “occupational radon”, “radon health effects”, “lung cancer”, “radon-induced diseases”, “biological effects”, “DNA damage”, “oxidative stress”, “bystander effect”, “inverse dose-rate effect”, “radon therapy”, “radon inhalation”, “radon bath”, “low-dose radon”, and “diffusing alpha-emitters radiation therapy (DaRT)”. These terms were searched individually or in combination using the Boolean operators AND and OR.

Publications were included if they addressed one or more topics relevant to this review, including the occurrence and sources of radon, human exposure pathways, detection and mitigation, epidemiological health effects, biological mechanisms, and potential therapeutic applications. Original research articles, epidemiological studies, experimental studies, reviews, meta-analyses, and relevant authoritative reports were considered. Additional relevant publications were identified through manual screening of the reference lists of key articles.

Duplicate records, publications unrelated to radon or outside the scope of this review, and studies that did not provide sufficient information relevant to the topics discussed were excluded. This article was designed as a comprehensive narrative review rather than a systematic review or meta-analysis. Accordingly, formal PRISMA-based study selection, standardized risk-of-bias assessment, and quantitative meta-analytic synthesis, including forest plots, were not performed.

## 3. Overview of Radon

### 3.1. Occurrence, Sources and Fate of Rn in Nature

#### 3.1.1. Discovery and Basic Properties of Rn

Radon (Rn), an inert gas with an atomic number of 86, was first discovered in 1900 by a German physicist Friedrich Ernst Dorn. Identified as a radioactive substance emitted from radium, radon and its progeny constitute a major source of natural radiation exposure, although their relative contribution varies among populations and regions. In Japan, for example, the estimated contribution from radon and thoron inhalation is substantially lower than the global average [18]. Radon has a density of approximately 9.9 g/L at 0 °C and 101.3 kPa, making it approximately 7.7 times denser than dry air under the same conditions [19]. The gas is colorless, odorless, and tasteless, which makes it difficult to detect without specialized equipment. The distribution and movement of radon in the natural environment are influenced by a combination of geological, atmospheric, and hydrological factors [20]. Geological composition, particularly in areas with elevated concentrations of uranium and thorium-key components of the Earth’s crust, plays a crucial role in radon generation [21]. As these elements decay, they release radon gas, which can migrate through soil and rock. The permeability of the soil and the presence of fractures or porous formations facilitate radon transport to the surface. Radon can accumulate in confined spaces, such as buildings, particularly in poorly ventilated indoor areas like basements [22]. Such accumulation is of concern due to its potential health risks associated with prolonged exposure.

#### 3.1.2. Source and Occurrence of Rn

**Figure 1:**
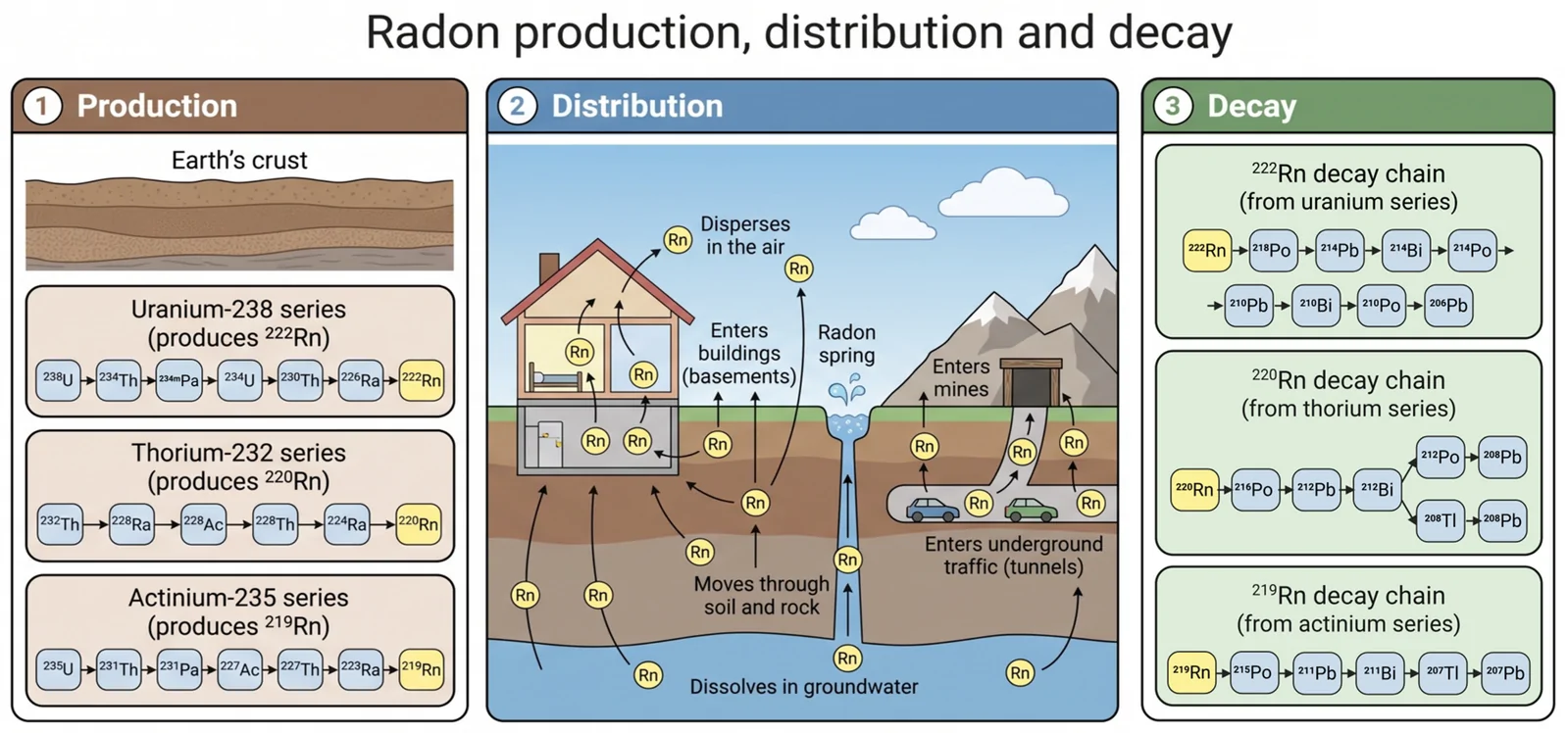
Radon production, distribution and decay. Arrows indicate the directions of radioactive decay and environmental transport. Colors are used to distinguish different radionuclides, decay series, and environmental components and do not represent quantitative values.

From uranium- and thorium-rich rocks in the Earth’s crust, radon can permeate natural matrix such as rocks, soil, and groundwater (Figure 1). Radon is an intermediate product in the decay chains of radioactive substances [23] and originates from three primary radioactive decay series: uranium (^238^U), thorium (^232^Th), and actinium (^235^U). These series, named after their parent isotopes, produce radon isotopes ^222^Rn, ^220^Rn and 219Rn, respectively, through successive decay processes [23]. Among them, thoron (^220^Rn) has a much shorter half-life (55 s) than ^222^Rn, limiting its migration, but it can still contribute to radiation exposure in thorium-rich environments. The widespread occurrence of radon in geological formations is primarily driven by the decay of uranium and thorium to radium, which subsequently produces radon. The principal decay chain of ^222^Rn proceeds through ^218^Po, ^214^Pb, ^214^Bi, ^214^Po, ^210^Pb, ^210^Bi, and ^210^Po, ultimately yielding stable ^206^Pb [23]. Minor decay branches are not listed here because they account for only a small fraction of the overall decay pathway. Radon has 27 known isotopes, among which four naturally occurring radioisotopes (^222^Rn, ^220^Rn, ^219^Rn and ^218^Rn) are of particular interest due to their relevance in environmental and health studies. Of these, ^222^Rn and ^220^Rn are particularly noteworthy due to their longer half-lives and substantial contribution to human radon exposure [24].

As radon undergoes spontaneous decay, it emits α-particles, leading to the formation of short-lived radioactive decay products. These decay products, including ^210^Pb, continue a longer-lived series that also emits α-particles, which can cause DNA damage at the cellular level. Although radon itself releases limited radiation, its short-lived progeny are the main contributors to the radiation dose [25]. The α-particles emitted during the decay of ^222^Rn are the dominant form of radiation, with β and γ emissions contributing minimally. Due to their limited penetration through matter, α-particles interact with nearby substances and pose significant risks to biological tissues when inhaled, potentially leading to cellular damage.

#### 3.1.3. Fate of Rn in Nature

Following its generation in geological materials, the environmental fate of radon is determined primarily by its mobility through porous media and its exchange among soil, groundwater, and the atmosphere. The extent of radon migration depends on factors such as soil permeability and moisture, groundwater flow, pressure gradients, and local geological conditions. These factors collectively influence the rate at which radon is transported from its source and accumulates in indoor or underground environments. The migration and transport of radon are driven by several processes, including diffusion and advective flow [26]. Diffusion occurs due to radon concentration gradients, while advective transport is influenced by pressure differences within soil and building foundations [27]. Atmospheric conditions, such as temperature, barometric pressure, and humidity, also affect radon behavior [20]. Additionally, the hydrological cycle impacts radon transport through groundwater [28]. In summary, radon distribution and transport are complex processes controlled by geological, atmospheric, and hydrological interactions.

### 3.2. Human Exposure Pathways

**Figure 2:**
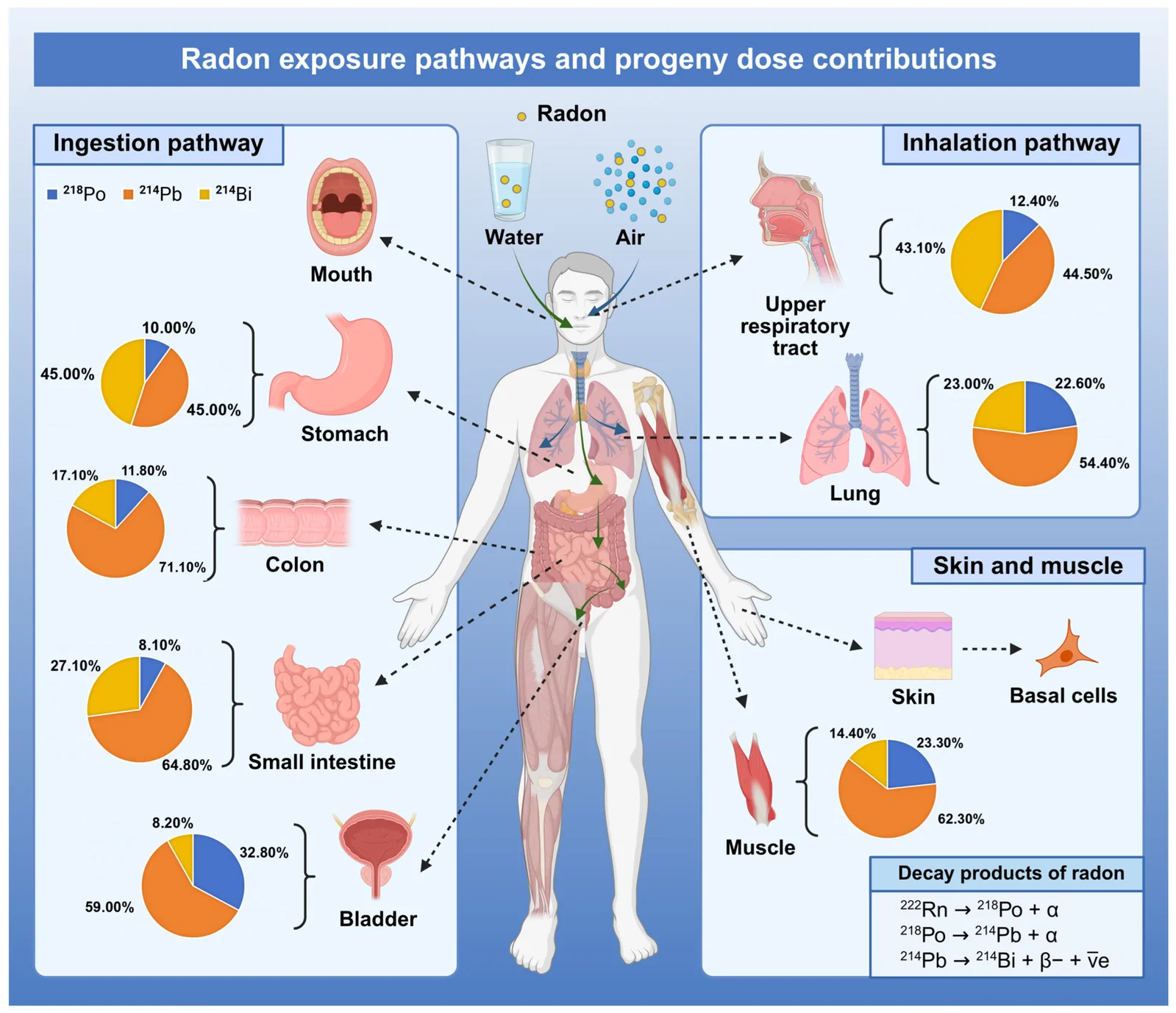
Radon exposure pathways and progeny dose contributions.

Radon can enter the human body through various pathways, including inhalation, ingestion, and dermal absorption. Kendall and Smith estimated organ and tissue doses resulting from the inhalation and ingestion of radon and its short-lived decay products using internal dosimetric models. Figure 2 was constructed from the organ-specific dose coefficients reported by Kendall and Smith [29], specifically from Tables 2 and 3 of that publication. For the inhalation pathway, the Type M results were used for particulate radon progeny; these calculations were based on the ICRP Human Respiratory Tract Model (ICRP Publication 66) [30] together with the relevant systemic biokinetic models. For the ingestion pathway, the values were derived from the dose coefficients reported under the assumption that radon decay products were in equilibrium. The pie charts show the relative contributions of ^218^Po, ^214^Pb, and ^214^Bi to the calculated dose within each selected organ or tissue. The percentages were normalized within each organ or tissue and therefore should not be used to compare absolute dose magnitudes among different organs. Thus, the distributions shown in Figure 2 reflect the combined influence of respiratory deposition, absorption and clearance, and systemic biokinetics, rather than the Type M absorption classification alone. Among these, inhalation is the most significant exposure route, particularly in indoor environments where radon and its progeny accumulate [31]. Once inhaled, radon and its progeny can be absorbed into the bloodstream, and it is estimated that about 1% of the inhaled radon is absorbed by the body [32]. Ingestion is another pathway, as radon can dissolve in water and be introduced into the body through drinking radon-containing water. While ingestion generally contributes less to overall radon exposure compared to inhalation, it is still a noteworthy route, particularly in regions where radon levels in water are high. Additionally, exposure can occur through dermal absorption, particularly during activities involving contact with radon-rich water, such as bathing in radon-containing hot springs [33]. While dermal absorption is a less significant pathway compared to inhalation and ingestion, it can contribute to overall exposure, especially in individuals who frequently use radon-rich water for therapeutic purposes.

As a radioactive gas, radon enters the human body primarily through inhalation, with the respiratory tract being the principal site of deposition and exposure [34]. After inhalation, a fraction of radon can cross the alveolar barrier and enter the bloodstream, allowing its transport to other tissues and organs. Because radon is relatively soluble in lipid-rich tissues, its distribution may include adipose tissue and bone marrow [35]. However, systemic circulation should be regarded primarily as a transport pathway rather than as a distinct anatomical organ of origin. In internal dosimetry models, radionuclides transferred through the blood are assigned to different source regions and compartments according to their biokinetic behavior, and the resulting absorbed dose depends on both radionuclide distribution and the spatial relationship between the source and target tissues [36]. Recent dosimetric analyses further demonstrated that heterogeneous radionuclide distribution and the microanatomical location of target cells can significantly influence local absorbed-dose estimates, particularly for short-range alpha particles [37]. Therefore, systemic circulation represents an important transport route for radon and its progeny, but the resulting radiation dose should be interpreted based on radionuclide-specific biokinetics and tissue microdistribution rather than assuming uniform exposure of all organs.

### 3.3. Detection of Radon

Advances in analytical techniques have led to the development of various methods for radon measurement, which can be broadly classified as passive and active techniques. Selecting an appropriate measurement method is essential for accurately determining radon concentrations and assessing the associated exposure risks [38].

One common and reliable method for radon detection is the use of ionization chambers. This technique is developed on the principle that radon and its decay products emit α- particles, which ionize the surrounding air molecules. The ionized particles create electron–ion pairs that, when subjected to an electric field, produce an electric current. This current is proportional to the concentration of radon in the air. Ionization chambers are highly sensitive and suitable for real-time monitoring. Research has demonstrated that ionization pulse chambers with double shielding structures are effective at detecting α-particle signals from radon decay, meeting industry standards for indoor radon concentration levels [39].

For long-term radon monitoring, alpha-track detectors (ATDs), also referred to as solid-state nuclear track detectors (SSNTDs), are among the most commonly used passive devices. These detectors typically employ materials such as CR-39 or LR-115, in which α-particles produce latent tracks that are subsequently revealed by chemical or electrochemical etching. Because they integrate radon exposure over extended periods, commonly from several months up to one year, ATDs are particularly suitable for estimating long-term average indoor radon concentrations [38,40]. Lucas scintillation cells, in contrast, are generally used for grab sampling or short-term radon measurements rather than as long-term passive dosimeters.

In general, the active detectors, such as scintillation detectors and ionization chambers, can provide rapid results within a period of one to seven days. These methods are useful for quick screenings, like those required during real estate transactions or to verify radon levels in newly constructed buildings. Therefore, they are usually used in short-term measurement. Long-term measurements, on the other hand, often employ passive detectors like nuclear track detectors, which measure radon over a period of several months. This extended monitoring period provides a more accurate estimate of the annual average radon concentration [41]. The choice of detection method depends on the required measurement duration and the sensitivity needed.

### 3.4. Activated Carbon for Radon Measurement and Removal

Activated carbon can be used as a passive medium for short-term radon measurement. Activated charcoal detectors adsorb radon from indoor air during a defined sampling period, typically several days, after which the collected activity is quantified by gamma counting or liquid scintillation methods. According to the WHO classification, activated charcoal detectors are primarily used for short-term measurements, generally over approximately 2–7 days, and their response can be influenced by factors such as humidity, temperature, and temporal variations in radon concentration [42].

Activated carbon adsorption can also be applied as a radon-removal technique, which should be distinguished from its use in passive measurement. In this context, activated carbon serves as an adsorbent for capturing radon from an air stream rather than as a dosimeter. Its adsorption capacity depends on factors including temperature, humidity, pore structure, and flow conditions. Static equilibrium, dynamic adsorption, and low-temperature adsorption systems have therefore been investigated for radon capture and removal [43,44,45,46]. Modifications such as low-temperature treatment can further enhance radon adsorption capacity [45], and activated-charcoal systems have been evaluated for radon and thoron removal applications [46].

For on-site radon evaluation, measurement strategies should be selected according to the monitoring objective and duration. Long-term measurements conducted over several months generally provide a more representative estimate of the annual average indoor radon concentration [38].

Additionally, developments in the modification of activated carbon, such as soaking in liquid nitrogen or employing evaporation techniques, have been shown to enhance its radon adsorption capacity by over 30% [45]. These modifications are relevant to radon capture and removal rather than to passive measurement. The applications of radon adsorption technologies are diverse, spanning from building safety assessments to occupational health protection in industries like mining and nuclear facilities, where radon levels must be closely monitored to ensure worker safety [46]. Overall, activated carbon has distinct roles in short-term radon measurement and in radon adsorption or removal, and these applications should be considered separately when selecting an appropriate method.

## 4. Radon-Induced Diseases

**Figure 3:**
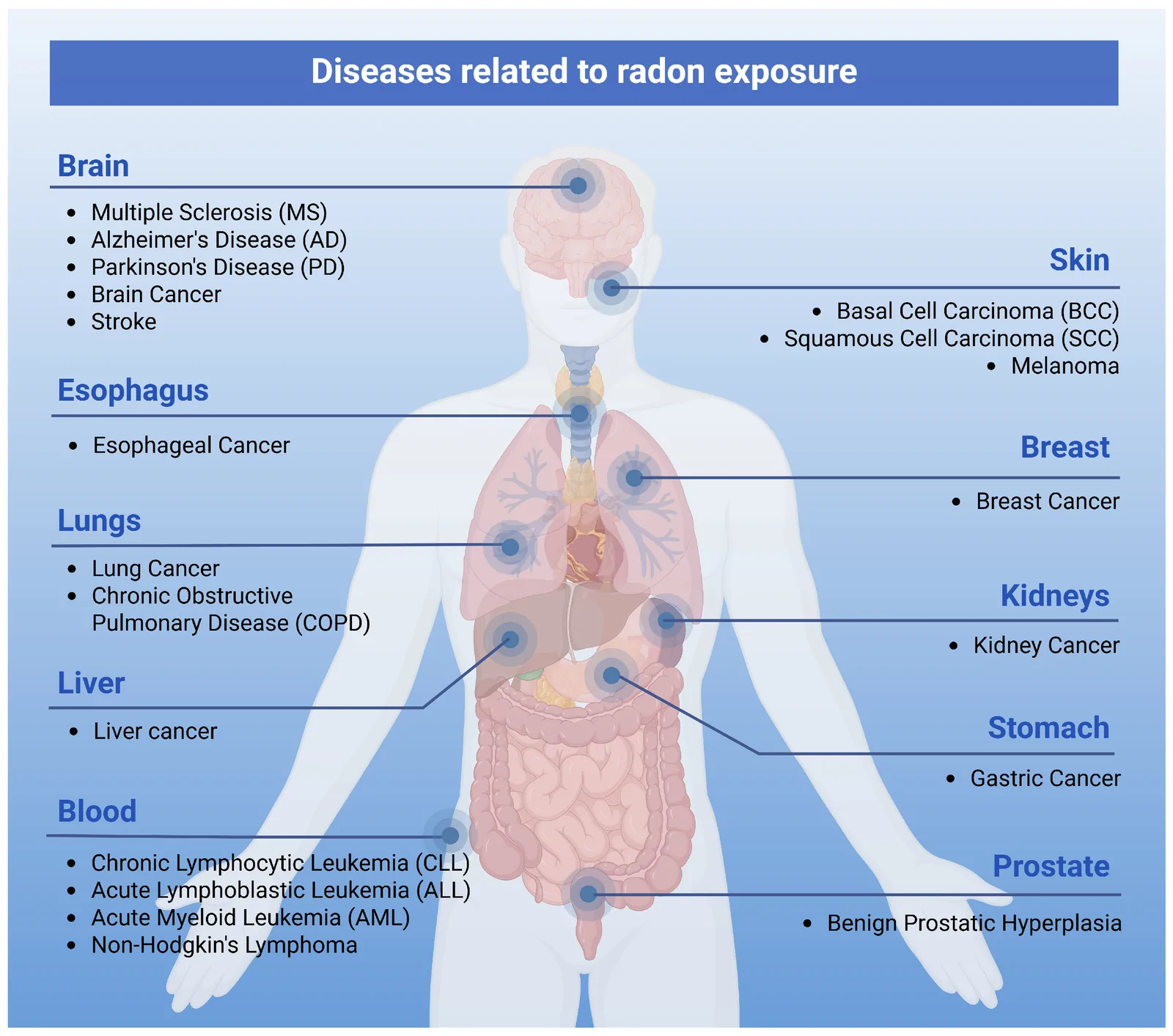
Diseases related to radon exposure.

Radon exposure is associated with various health conditions, primarily affecting the respiratory system but also impacting other organs. The most well-documented disease linked to radon is lung cancer, where α-particle radiation induces DNA damage, genomic instability, and malignant transformation of lung cells. Possible associations with COPD, hematological disorders, skin diseases, and other systemic effects have also been investigated (Figure 3), although these relationships remain less well established and require further study.

### 4.1. Respiratory Diseases

#### 4.1.1. Lung Cancer

Lung cancer is the best-established health outcome associated with radon exposure and remains a leading cause of cancer-related mortality worldwide. According to the WHO, radon is the second leading cause of lung cancer after smoking. Radon exposure, particularly from inhalation of its progeny, leads to the deposition of radioactive particles in the lungs, causing DNA damage that can lead to gene mutations and carcinogenesis.

The contribution of radon exposure to lung cancer varies geographically. It is estimated that radon exposure contributes to 3% to 14% of all lung cancer cases in various countries [38]. In Europe, radon exposure is estimated to account for about 9% of lung cancer deaths and 2% of overall cancer deaths [47]. A cross-Canada survey found that 14–16% of lung cancer deaths among Canadians are attributable to indoor radon exposure [48]. These variations may reflect differences in residential radon concentrations, housing characteristics, population smoking patterns, and exposure assessment methods.

Radon and tobacco smoking interact to substantially increase the absolute risk of lung cancer. In the pooled European analysis, the absolute risk of lung cancer was estimated to be approximately 25 times higher among cigarette smokers than among lifelong non-smokers at comparable residential radon concentrations. More importantly, pooled residential epidemiological studies have demonstrated a clear exposure–response relationship between radon concentration and lung cancer risk. A collaborative analysis of 13 European case–control studies reported an 8.4% increase in lung cancer risk per 100 Bq/m^3^ increase in measured residential radon concentration, which increased to approximately 16% after correction for uncertainties in estimating long-term exposure. The exposure–response relationship was consistent with a linear model, with no evidence of a threshold [49]. A pooled analysis of seven North American case–control studies reported a broadly comparable increase of approximately 11% per 100 Bq/m^3^ [50].

Evidence from never-smokers further supports this association. In a Swedish case–control study comprising 436 never-smoking lung cancer cases and 1649 never-smoking controls, the excess relative risk was estimated to increase by approximately 10% per 100 Bq/m^3^ increase in average residential radon concentration [51]. These findings further support an association between residential radon exposure and lung cancer risk among never-smokers, indicating that the observed association is not restricted to smokers.

Occupational studies provide complementary evidence at higher cumulative exposures. Elevated lung cancer risks have been reported among workers in underground environments exposed to radon and its progeny [52]. The Wismut cohort, one of the largest uranium miner cohorts worldwide and comprising approximately 58,700 miners, demonstrated an exposure-related increase in lung cancer risk [53]. Lubin et al. pooled data from 11 cohort studies of radon-exposed underground miners and confirmed an inverse dose-rate effect, whereby, for a given cumulative exposure, lung cancer risk per unit exposure tended to be greater at lower exposure rates and longer exposure durations; however, this effect appeared to diminish at low cumulative exposures [54].

Collectively, evidence from residential and occupational studies indicates that lung cancer risk increases with cumulative radon exposure, while the magnitude of risk is modified by factors such as smoking status, exposure duration, and exposure rate. These observations emphasize the importance of long-term radon measurement and mitigation in both residential and occupational environments [2].

Despite the substantial epidemiological evidence linking radon exposure to lung carcinogenesis, several uncertainties remain, particularly regarding prolonged low-level exposure and risks among never-smokers and other susceptible populations. Future studies should refine exposure–response estimates, improve long-term exposure assessment, and clarify interactions among radon, smoking, genetic susceptibility, and other environmental factors. These efforts will help strengthen risk assessment and inform more effective mitigation strategies.

#### 4.1.2. Chronic Obstructive Pulmonary Disease (COPD)

COPD is a progressive respiratory disease characterized by persistent airflow limitation and chronic airway inflammation, with cigarette smoking remaining its predominant established risk factor. Residential radon exposure has also been investigated as a potential contributor to COPD morbidity and mortality, although the available epidemiological evidence remains limited and heterogeneous [55].

Evidence from the Cancer Prevention Study II conducted in the United States reported a significant association between residential radon exposure and COPD mortality, with a hazard ratio of 1.13 per 100 Bq/m^3^ increase in radon concentration (95% CI: 1.05–1.21) [56]. Additionally, women appear to be more vulnerable to radon-associated COPD, as elevated radon exposure has been linked to increased hospitalizations in this group [57]. However, some studies have indicated that radon exposure does not significantly affect the clinical characteristics of COPD in current or former smokers. Furthermore, no clear trend has been observed in the decline of forced expiratory volume in one second (FEV1) with increasing residential radon exposure [58]. These findings highlight the need for further research to elucidate the potential role of radon exposure in COPD pathogenesis and progression.

### 4.2. Hematological Diseases

The relationship between radon exposure and hematological diseases, particularly leukemia and lymphoma, has yielded conflicting findings. Some studies suggest a potential positive correlation. For example, radon exposure has been associated with an increased risk of chronic lymphocytic leukemia (CLL), with mechanisms possibly analogous to those implicated in lung cancer development [59]. A Danish study involving 2400 cases of leukemia found that domestic radon exposure elevated the risk of acute lymphoblastic leukemia (ALL) in children, but not other childhood cancers [60]. In Canada, a significant correlation was reported between indoor radon levels and acute myeloid leukemia (AML) incidence [60]. Additionally, Korean research indicated that higher indoor radon levels increased the risk of non-Hodgkin’s lymphoma and other hematological malignancies, particularly among younger individuals [61]. A U.S. cohort study found that women living in regions with elevated radon levels had a higher risk of developing lymphoid malignancies [12].

However, other studies have failed to substantiate these associations. A large-scale Canadian survey spanning 33 metropolitan areas did not find a statistically significant relationship between city-level radon concentrations and risks of leukemia or lymphoma in children aged 0–14 [60]. Similarly, research on Eldorado uranium miners found no connection between radon decay product exposure, γ-ray doses, and hematological cancer incidence or mortality [62]. European cohort studies involving over 700,000 children found no association between domestic radon exposure and childhood leukemia after adjusting for confounders [63]. These findings align with dose estimates indicating that radon exposure has minimal impact on red bone marrow [64].

In summary, while some evidence suggests a possible link between radon exposure and hematological diseases, other studies contradict these findings. This discrepancy underscores the need for further research, particularly to clarify the role of confounding factors and population-specific risks.

### 4.3. Skin Disease

The potential association between radon exposure and skin cancer has been investigated in several studies, although the overall evidence remains inconclusive. Ultraviolet (UV) radiation is the major established risk factor for skin cancer, whereas a possible contribution from residential radon exposure has been suggested in some epidemiological studies [65].

Several studies have examined the association between radon exposure and basal cell carcinoma (BCC), squamous cell carcinoma (SCC), and melanoma. For example, research conducted in geothermal regions, such as Iceland, reported an elevated risk of BCC among residents exposed to high radon levels [66]. However, these findings were complicated by significant UV exposure and other environmental factors in these areas, making it challenging to isolate radon’s effects. A large Danish cohort study involving over 51,000 participants found a potential link between residential radon exposure and BCC, with a 13.6-year follow-up period revealing a modest increase in risk [65]. The association between radon exposure and SCC has also been explored. An ecological study in southwest England found higher SCC incidence rates in regions where average radon levels exceeded 230 Bq/m^3^ compared to areas with lower levels [13]. The results of another nationwide prospective cohort study suggest that residential radon exposure is a relevant risk factor for melanoma even when taking long term-average residential UV exposure into account [67]. These findings align with national cohort studies suggesting that radon exposure may act as an independent risk factor for melanoma and other skin cancers, even when long-term UV exposure is accounted for [13].

Mechanistically, radon decay products emit α-particles, which deposit on the skin’s surface [14]. In most cases, the outer layer of dead skin absorbs this energy, limiting biological harm. However, in areas with thinner skin, such as the face, α-particles may reach sensitive basal cells, potentially contributing to carcinogenesis.

In conclusion, while studies suggest a potential association between radon exposure and certain skin cancers, the findings are not definitive due to confounding factors like UV exposure and individual susceptibility. Further research is essential to clarify the role of radon in skin carcinogenesis and to better understand its interaction with other environmental and genetic factors.

### 4.4. Digestive Diseases

Radon exposure has been investigated as a possible risk factor for digestive system malignancies, particularly gastric cancer; however, epidemiological findings remain inconsistent and a causal association has not been established [68].

Several studies provide evidence supporting the hypothesis that radon exposure increases the incidence of digestive cancers. For example, an ecological study in North Carolina found that after controlling for confounding factors and spatial autocorrelation, radon exposure in groundwater was associated with a 24% (OR = 1.24, 95% CI 1.03–1.49) increase in gastric cancer case clustering [69]. Another retrospective study in Pennsylvania showed a positive correlation between radon levels and gastric cancer incidence in women (*p* < 0.05), as well as gastric cancer mortality in both sexes (*p* < 0.05), suggesting that radon exposure might contribute to gastric cancer mortality [70]. Furthermore, a prospective cohort study found a significant and strong association between residential radon exposure and gastric cancer incidence, with a risk ratio of 10.8 (95% CI 1.2–98.0), although the study’s small sample size and limited evidence warrant further investigation [71]. An ecological study in Spain’s Galicia region also reported a statistically significant association between residential radon exposure and male esophageal cancer mortality (*p* < 0.001), while no such relationship was observed in females, suggesting that radon exposure might be a risk factor for male esophageal cancer mortality [72].

On the other hand, some studies did not find a significant association between radon exposure and digestive cancers. A review of 13 cohort studies across Europe, North America, and Japan found no significant link between radon exposure and oral or pharyngeal cancer. However, due to study limitations and indirect exposure assessments, further long-term research in high-radon areas is needed [73]. For instance, a cohort study conducted in Finland, involving 144,627 residents using groundwater and well water, found no significant relationship between radon, ^226^Ra, or natural uranium concentrations in drinking water and gastric cancer risk. The hazard ratios for radon, ^226^Ra, and uranium were 0.68 (95% CI 0.29–1.59), 0.69 (95% CI 0.33–1.47), and 0.76 (95% CI 0.48–1.21), respectively, indicating no increased gastric cancer risk at these exposure levels [74]. Similarly, a historical cohort study in Misasa Town, Japan, compared cancer incidence and mortality between high-radon and low-radon areas. Despite a downward trend in gastric cancer incidence among both men (RR = 0.70, 95% CI 0.44–1.11) and women (RR = 0.58, 95% CI 0.34–1.00), the results were not statistically significant. Moreover, the study did not measure individual exposure levels or control for important confounders like smoking and diet, which limits the interpretability of the findings [75].

In conclusion, while several studies support the hypothesis that radon exposure may be linked to an increased risk of digestive system cancers, particularly gastric and esophageal cancers, the overall evidence remains inconsistent. The differences in study findings, along with the methodological limitations in some studies, underscore the need for further high-quality, large-scale, and long-term research to better understand the potential carcinogenic effects of radon exposure on digestive health.

### 4.5. Other Diseases

In addition to the diseases mentioned above, researchers have explored the potential links between Rn exposure and other diseases. For instance, studies are investigating the relationship between radon gas and neurodegenerative conditions such as multiple sclerosis (MS), Alzheimer’s disease (AD), and Parkinson’s disease (PD) [76]. Preliminary findings suggest that radon may indirectly contribute to the pathogenesis of these disorders by inducing oxidative stress and inflammatory responses.

## 5. Biological Mechanisms of Radon-Induced Diseases

**Figure 4:**
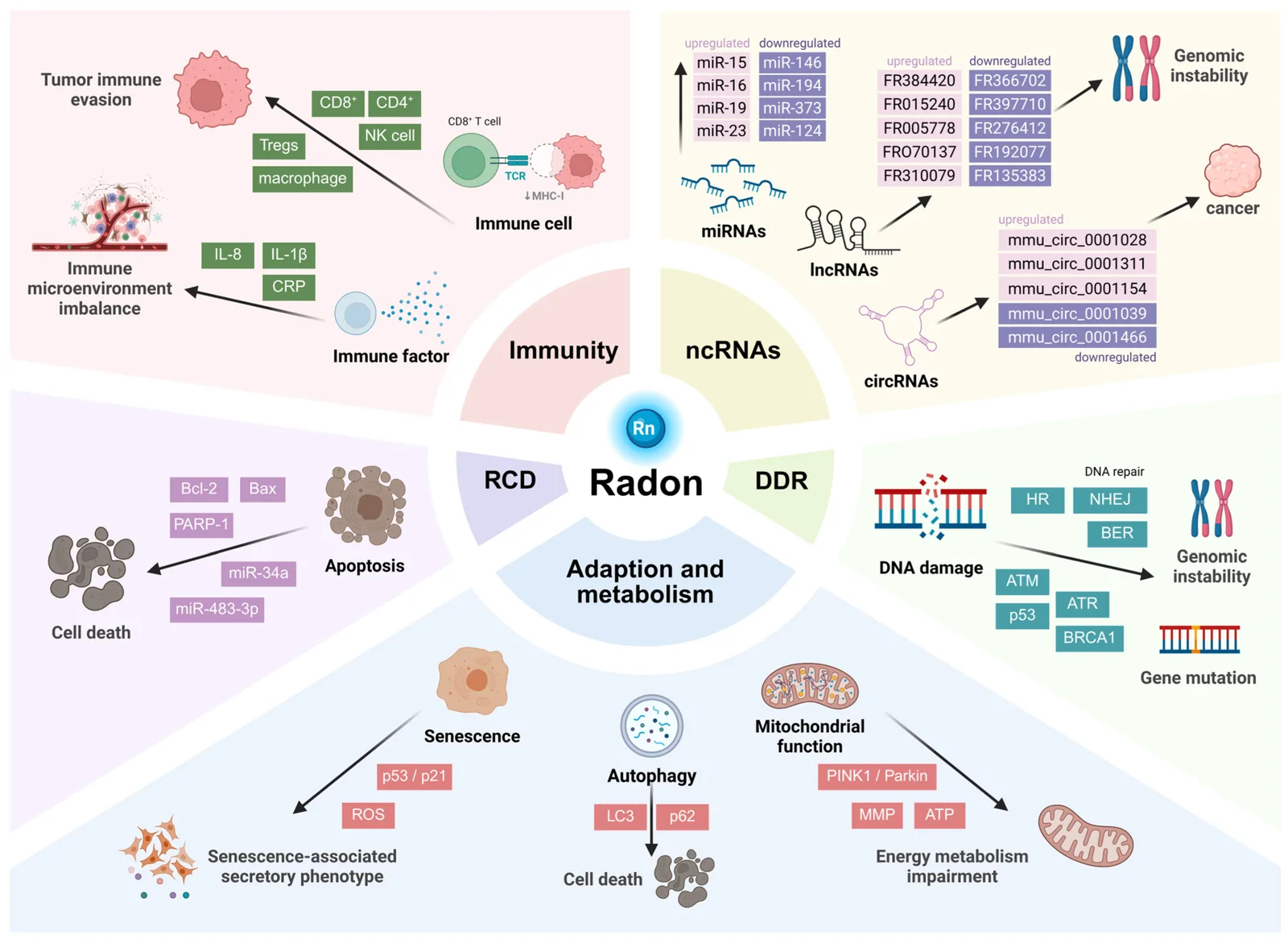
Biological mechanisms of radon-induced cancer. Arrows indicate the direction of the indicated biological effects; ↓ indicates downregulation.

Radon exposure triggers a cascade of biological alterations across multiple levels, ultimately promoting disease development (Figure 4). Histopathologically, it induces inflammation, vascular injury, pulmonary fibrosis, and various subtypes of lung cancer. At the cellular and subcellular levels, radon affects cell cycle progression, apoptosis, autophagy, senescence, EMT, immune regulation, and mitochondrial activity [77]. On the molecular level, it causes DNA damage, chromosomal instability, protein and epigenetic modifications, and disrupts intracellular signaling pathways. Specific radiation-related phenomena such as the bystander effect (BE), enhanced deposition effect (EDE), and inverse dose-rate effect (IDRE) further amplify these changes [78]. In addition, radon may influence microbial communities, potentially altering host–microbiota interactions [79].

### 5.1. Histopathological Alteration

Chronic radon exposure leads to notable pathological changes in lung tissue, including inflammation, vascular injury, fibrosis, and multiple histological subtypes of lung cancer [78]. The emitted α-particles directly injure tissues and induce oxidative stress, attracting inflammatory cell infiltration and promoting the release of cytokines that sustain a chronic inflammatory milieu [80]. This persistent inflammation hampers normal tissue repair, resulting in abnormal collagen accumulation and progressive fibrosis, ultimately impairing lung function [77]. In parallel, radon-induced cellular and genetic damage contributes to malignant transformation and is strongly associated with distinct lung cancer pathologies [78].

#### 5.1.1. Inflammatory Response

Radon exposure induces tissue damage primarily through α-particles, which trigger oxidative stress and stimulate airway epithelial cells to release inflammatory mediators such as IL-8, IL-1β, and C-reactive protein [11,80]. These mediators recruit neutrophils and macrophages, forming a localized inflammatory microenvironment [81]. Animal studies have shown that radon inhalation leads to alveolar structural disruption, septal rupture, interstitial thickening, and bronchial alterations, ultimately impairing pulmonary function [82,83,84].

While radon concentrations in the bloodstream remain low [85], its decay products may still elicit systemic inflammatory responses. Chronic inflammation not only damages tissues but also promotes tumorigenesis. For instance, radon-induced IL-8 elevation is linked to COPD and lung cancer progression [58]. Sustained inflammation can also drive angiogenesis by upregulating factors such as VEGF, thereby facilitating a tumor-permissive microenvironment [86]. Thus, radon-induced inflammation plays a dual role in tissue injury and cancer development.

#### 5.1.2. Vascular Damage

Radon exposure contributes to vascular injury through endothelial damage, arterial fibrosis, and lipid metabolism disturbances. Inhaled radon can harm vascular endothelial cells, which are essential for maintaining vascular integrity and regulating blood flow. Damage to these cells increases permeability, allowing inflammatory mediators and leukocytes to infiltrate vessel walls, thereby promoting inflammation and the development of atherosclerosis [87,88].

Radon-induced radiation may also trigger arterial fibrosis, leading to reduced vascular elasticity and impaired blood flow, heightening the risk of cardiovascular diseases [5]. In addition, α-particles can oxidize low-density lipoproteins (LDL), which further intensify vascular inflammation and promote atherosclerotic plaque formation through macrophage activation and platelet aggregation [76,87].

Collectively, these findings indicate that radon-related vascular damage extends beyond localized effects, potentially threatening systemic circulatory health.

#### 5.1.3. Pulmonary Fibrosis

Chronic radon exposure can lead to pulmonary fibrosis, marked by excessive collagen buildup in lung tissue, reduced elasticity, and impaired respiratory function [89]. This process often occurs alongside chronic inflammation, which further accelerates lung damage [90]. As early as 1996, Archer et al. reported the first case linking radon progeny exposure to pulmonary fibrosis [22]. Subsequent studies using animal models confirmed that radon, alone or with uranium dust, could induce fibrosis and emphysema [91]. More recent research highlights oxidative stress and inflammation as key drivers of this condition [77].

Oxidative stress from excess ROS damages lung cells and promotes fibroblast activation and collagen deposition, contributing to fibrosis [77,92]. Antioxidants have shown some protective effects in reducing this damage [92]. In parallel, chronic inflammation triggered by radon exposure promotes immune cell infiltration and fibroblast proliferation, exacerbating fibrotic lesions. Additionally, repeated exposure may induce epithelial–mesenchymal transition (EMT), enabling epithelial cells to acquire fibroblast-like traits, further promoting fibrosis [77].

Together, these findings suggest that oxidative stress, inflammation, and EMT play central roles in radon-induced pulmonary fibrosis, offering insight into its prevention and management.

#### 5.1.4. Pathological Types of Lung Cancer

Lung cancers associated with radon exposure encompass both small-cell lung cancer (SCLC) and non-small-cell lung cancer (NSCLC), with pathological features varying across histological subtypes. Radon-associated tumors have been reported to exhibit morphological abnormalities, including nuclear enlargement, cellular atypia, and disruption of normal tissue architecture [93].

SCLC, known for its aggressive nature and rapid growth, is characterized histologically by small cells with scant cytoplasm, high mitotic activity, and a strong tendency toward local invasion and early metastasis [94]. Rather than reiterating its epidemiological association with radon exposure, the pathological significance of SCLC in this context lies in its highly aggressive phenotype.

NSCLC, the most common form of lung cancer, includes subtypes such as squamous cell carcinoma and adenocarcinoma. Squamous cell carcinoma typically originates in the bronchial epithelium and is associated with keratinization and squamous metaplasia. Adenocarcinoma generally arises in peripheral lung tissue and is characterized by glandular differentiation, and it is frequently observed in non-smokers. Large cell lung cancer represents a less common NSCLC subtype and has been comparatively less characterized in relation to radon-associated pathological changes.

Overall, available studies suggest that radon-associated lung cancers are not restricted to a single histological subtype, although the reported distribution of SCLC, squamous cell carcinoma, and adenocarcinoma varies among study populations [93,95,96]. Such variability may reflect differences in smoking status, exposure level, population characteristics, and study design. Further pathological and molecular characterization is needed to determine whether radon exposure is associated with distinct tumor phenotypes or subtype-specific biological features.

### 5.2. Cellular and Subcellular Phenotypes Induced by Radon

Radon exposure elicits diverse cellular and subcellular changes that underlie its pathological effects. It disrupts cell cycle regulation and proliferation, contributing to aberrant growth or impaired repair. Stress responses such as apoptosis, autophagy, and senescence determine cell survival under radon-induced damage [97]. Radon also promotes EMT and reshapes the immune microenvironment, facilitating tissue remodeling and tumor progression [77]. Mitochondrial dysfunction, particularly oxidative stress and altered metabolism, further amplifies these effects [98]. Together, these interrelated processes drive the complex biological consequences of radon exposure.

#### 5.2.1. Cell Cycle and Cell Proliferation

Radon exposure disrupts normal cell cycle regulation by altering gene and protein expression. α-particle radiation can upregulate cell cycle inhibitors such as *CDKN1A* and *GADD45A*, while downregulating DNA replication-related genes like the *MCM* family, ultimately leading to cell cycle arrest [99]. DNA damage from radon, including double-strand breaks and point mutations, may also induce epigenetic changes; for instance, increased *CDKN2A* promoter hypermethylation has been observed in radon- or plutonium-exposed lung cancer cases [100].

Protein-level alterations further support these findings. In radon-exposed miners, increased expression of CDKs and cyclins (e.g., CDK2, CyclinD1) suggests disrupted cycle progression [101]. Radon-induced DNA damage also leads to a dose-dependent increase in micronuclei in lung fibroblasts, reflecting mitotic errors and potential G2/M arrest [102].

Radon activates the p53 and ATM pathways, impairing mitochondrial function and triggering cell cycle checkpoints. These effects include reduced ATP, elevated ROS, increased mtDNA copy number, and enhanced G2/M arrest, alongside EMT-like phenotypes [97,98]. Additionally, radon exposure alters microRNA expression, influencing pathways related to oxidative stress, inflammation, and oncogenesis [103]. Repeated exposure may also promote epithelial cell migration and proliferation while reducing adhesion, contributing to increased invasiveness and malignant transformation [77].

#### 5.2.2. Apoptosis

Radon exposure affects apoptosis through oxidative stress, mitochondrial dysfunction, DNA damage response, and epigenetic regulation. The accumulation of ROS following radon exposure damages cellular structures and promotes apoptosis [104]. Mitochondrial injury contributes by disrupting energy metabolism [105], though paradoxically, some studies report increased mitochondrial function, elevated membrane potential, and mtDNA copy number, which may support proliferation and reduce apoptosis alongside EMT-like changes [10]. Melatonin has been found to partially reverse these mitochondrial alterations and mitigate oxidative stress [106].

Apoptotic responses vary depending on mitochondrial integrity. In human bronchial epithelial cells, radon exposure increases apoptosis, but cells lacking functional mitochondria (ρ−) show reduced apoptotic levels compared to normal (ρ+) cells [104]. The redistribution of cell cycle phases, particularly G2/M arrest, is more pronounced in ρ+ cells and may influence transformation.

Radon also modulates apoptosis-related genes. In MCF-7 cells, low-dose α-radiation downregulates *bax* and *bcl-2*, while upregulating *Bcl-xL* and *Bcl-xS*, sensitizing cells to apoptosis and potentially enhancing chemotherapy efficacy [107]. Changes in miRNA expression (e.g., miR-34a, miR-125b, miR-18a) affect signaling pathways like MAPK, Wnt, and NF-κB, regulating both apoptosis and malignant transformation [84,108]. miR-34a, in particular, promotes apoptosis by increasing Bax and reducing Bcl-2 and PARP-1 in BEAS-2B cells [109].

Low-dose radon exposure may also activate protective epigenetic mechanisms. Through stabilization of p53 and modulation of DNA methylation, radon influences expression of tumor suppressors such as *VHL*, *CDKN2A*, and *MLH1*, contributing to a selective removal of abnormal cells via epigenetic apoptosis (epiapoptosis) [110]. Additionally, DNA double-strand breaks induced by α-particles activate ATM kinase, leading to DDR, p38MAPK/Bax-mediated apoptosis, autophagy, and cell cycle arrest [97].

#### 5.2.3. Autophagy

Autophagy is a cellular process that removes damaged organelles and proteins, contributing to stress adaptation and homeostasis. Radon exposure induces DNA double-strand breaks (DSBs) and activates ATM kinase, which is a key regulator of both DNA repair and autophagy [97]. This activation initiates autophagy as a protective response to mitigate damage and support cell survival [97,111].

In bronchial epithelial cells, repeated radon exposure triggers sustained ATM-mediated autophagy, helping eliminate damaged cellular components [97]. However, with prolonged or high-dose exposure, this mechanism may become impaired, reducing its protective effect. Environmental levels of radon have been shown to disrupt autophagy, leading to accumulated damage, increased ROS, and endoplasmic reticulum stress [105,112]. This suggests that while autophagy initially protects against radon-induced injury, its dysregulation may contribute to long-term cellular dysfunction.

#### 5.2.4. Cellular Senescence

Cellular senescence is a state of permanent cell cycle arrest triggered by stressors such as DNA damage, oxidative stress, and inflammation. While it contributes to aging, it also plays roles in cancer and age-related diseases. Radon exposure has been shown to promote senescence through multiple pathways. The α-particles from radon decay cause DNA double-strand breaks, activating the DNA damage response and leading to senescence-associated growth arrest [113,114]. In parallel, radon-induced oxidative stress damages cellular macromolecules, further accelerating senescence [115,116]. Additionally, radon exposure may provoke local and systemic inflammation, and inflammatory cytokines are known drivers of cellular senescence and tissue aging [78,116].

#### 5.2.5. Epithelial–Mesenchymal Transition (EMT)

Radon exposure has been implicated in inducing EMT, a process where epithelial cells acquire mesenchymal traits, gaining enhanced motility and invasiveness. This transition involves reduced expression of adhesion molecules like E-cadherin and increased levels of mesenchymal markers such as N-cadherin, Vimentin, and transcription factors SNAI1/2 and ZEB1/2, facilitating cell detachment and migration [10]. Long-term radon exposure alters mitochondrial function, enhancing oxidative phosphorylation and antioxidant capacity, which may alleviate ROS accumulation while promoting EMT-like changes. In BEAS-2B cells, radon induces EMT along with metabolic reprogramming, including reduced ATP levels, increased ROS, mtDNA copy number changes, and G2/M arrest. These shifts are accompanied by increased expression of TIGAR and p53R2 and decreased SCO2, suggesting a shift from oxidative phosphorylation to glycolysis. In mouse lung tissue, radon also upregulates p53-related proteins, indicating a stress response that may initially protect cells but ultimately contributes to malignant transformation [98].

#### 5.2.6. Immune Microenvironment

The immune microenvironment comprises immune cells, cytokines, chemokines, and extracellular components interacting with tumor cells. Radiation can disrupt its homeostasis, not only by directly affecting immune cells but also by altering extracellular matrix components and signaling molecules, thereby impairing immune function and increasing tissue toxicity.

Radon exposure modulates the immune microenvironment through oxidative stress, immunosuppression, inflammation, and reduced immune surveillance, collectively weakening the body’s defense against disease [77]. It also enhances epithelial cell proliferation and migration, partly through the loss of normal cell cycle control following DNA damage. These changes promote uncontrolled growth and may initiate tumorigenesis.

Apoptosis, typically activated in response to DNA damage, may be bypassed in radon-exposed tumor cells, enabling their continued survival [53]. Moreover, radon may induce immune escape by altering tumor antigen expression and promoting an inflammatory milieu through cytokine release, further shaping a tumor-promoting microenvironment [77,89].

Radon exposure affects both the quantity and functionality of immune cells. In mice, it reduces cellularity in lymphoid organs such as the thymus and peripheral lymph nodes. Specifically, it decreases CD4^+^ CD8^+^ thymocytes and increases immature CD4^−^ CD8^−^ T cells, while altering the balance between T and non-T cells [117]. In lung-draining lymph nodes, T cell mitogen responses are suppressed, whereas B cell responses remain largely unaffected. These effects may result from α-particle-induced cytotoxicity or altered expression of migration and adhesion molecules like CD3 and CD44. Conversely, radon enhances mitogen-induced proliferation of T and B cells in the spleen and peripheral lymph nodes, suggesting complex, tissue-specific effects on immune function [117]. Additionally, radon may reduce the activity of cytotoxic T cells and natural killer (NK) cells, compromising tumor surveillance and antiviral immunity [118].

Radon exposure exerts distinct immunological effects depending on dose. Low-dose radiation can subtly enhance immune activity, including cytokine production, immune cell proliferation, and intercellular signaling, potentially contributing to its therapeutic value in autoimmune conditions [119]. In contrast, high-dose exposure predominantly causes immunosuppression, through direct cytotoxicity to immune cells and disruption of signaling pathways.

A proposed mechanism for radon therapy involves activation of antioxidant defenses. Inhaled radon may stimulate endogenous antioxidant systems, reducing oxidative damage and preserving immune cell function [14]. However, prolonged exposure may impair immune surveillance by decreasing immune cell numbers and functionality, increasing susceptibility to infection and cancer.

Radon also alters immune regulation, affecting the Th17/Treg balance. While Treg cells suppress excessive immune responses, Th17 cells drive inflammation; radon-induced shifts in this balance may modify overall immune reactivity [120]. In mice, radon exposure slows weight gain, reduces serum lipids, and diminishes immune cell diversity, indicating broad effects on immune homeostasis [79].

Within the lung, radon may impair macrophage recruitment and weaken the pulmonary immune response [121]. Even short-term exposure has been linked to immune suppression, potentially creating a microenvironment conducive to tumor growth and metastasis [122].

Radon exposure induces excessive reactive oxygen species (ROS) production in the lungs, leading to oxidative stress that damages pulmonary cells and impairs immune cell function [53]. This disruption can dysregulate immune responses, increasing vulnerability to infections and tumor development. Elevated oxidative stress may also drive overproduction of inflammatory cytokines, contributing to chronic inflammation that affects not only the lungs but also distant organs, compromising overall health [53].

Radon can also interfere with immune regulation by disrupting signaling pathways that normally maintain immune homeostasis. Inhalation of radon alters cytokine secretion, particularly enhancing Th1-type responses. Increased levels of Th1 cytokines suggest a shift toward cell-mediated immunity, which may improve pathogen clearance [123]. However, other studies report that radon suppresses both Th1 and Th17 cytokine production, potentially alleviating chronic inflammation [16]. These conflicting findings underscore the need for further research to clarify the long-term effects of radon on immune balance and identify strategies to reduce its health risks.

#### 5.2.7. Mitochondria-Related Mechanisms

Radon exposure impairs mitochondrial function through multiple interrelated mechanisms, contributing to oxidative stress, genomic instability, and malignant transformation. Damage to mitochondrial DNA (mtDNA), a key component of energy metabolism, has been observed in radon-exposed bronchial epithelial cells, leading to decreased mitochondrial membrane potential, elevated ROS, and disrupted cell cycle distribution [104]. These alterations promote tumorigenesis, particularly when combined with nuclear DNA damage. Chronic exposure may further compromise DNA repair, resulting in irreversible mutations.

Circulating free mitochondrial DNA (cf-mtDNA), released during cellular stress or apoptosis, increases significantly following radon exposure. These cf-mtDNA fragments can activate immune signaling and amplify lung inflammation, exacerbating cancer progression [124]. Elevated cf-mtDNA levels have been reported in various malignancies, including radon-associated lung cancer, suggesting potential as an early diagnostic biomarker [125]. However, its mechanistic role in radon-induced carcinogenesis remains to be fully clarified [124].

Radon-induced ROS also affect microRNAs (miRNAs), which regulate gene expression both in the nucleus and mitochondria. Altered miRNA profiles under radon stress may disrupt energy metabolism and mitochondrial stability by targeting key metabolic genes [103,126]. Furthermore, changes in miRNA localization and abundance may impair cellular stress responses and promote mitochondrial dysfunction.

Radon decay emits α-particles that deposit ionizing radiation into lung tissues, initiating cellular damage primarily through the overproduction of ROS [104]. Elevated ROS levels compromise mitochondrial membranes and the electron transport chain, reduce mitochondrial membrane potential, and ultimately trigger cell death [127]. In parallel, ROS oxidize macromolecules such as lipids, proteins, and nucleic acids, contributing to cellular dysfunction and genomic instability, a key driver of carcinogenesis [122,128]. Persistent oxidative stress can disrupt apoptosis, alter the cell cycle, and promote unchecked proliferation, thereby facilitating malignant transformation [129,130].

Long-term radon exposure also induces the formation of mitochondrial permeability transition pores, which heighten membrane permeability and further exacerbate mitochondrial injury [130]. Interestingly, in certain chronically exposed populations, such as residents of Tande-Tande sub-village in Indonesia, reduced micronuclei formation and lower catalase levels suggest a possible radiation adaptation response (RAR), indicating altered oxidative stress management under prolonged exposure [131].

In addition to mitochondrial impairment, ROS generated by radon activate matrix metalloproteinases (MMPs), a group of enzymes involved in extracellular matrix remodeling. Upregulation of specific MMPs under oxidative stress enhances tumor cell migration and invasion, playing a critical role in metastasis [77,132]. Simultaneously, radon-induced immune suppression and molecular protein alterations reshape the tumor microenvironment, increasing the complexity of lung cancer pathogenesis [104]. Apoptotic pathways may also be activated as a compensatory response, yet the resulting cell death can amplify mitochondrial damage if regulation is impaired [133].

Moreover, mitochondrial autophagy (mitophagy) emerges as a cellular defense mechanism against radon-induced damage. Studies in BEAS-2B cells and murine lung tissue have shown that prolonged exposure disrupts redox balance and induces progressive mitochondrial degradation. Activation of the Nrf2 pathway and its downstream antioxidants, alongside altered expression of mitophagy markers such as *PINK1*, *Parkin*, and *p62*, suggest that Nrf2 functions as a central regulator in maintaining mitochondrial integrity and mitigating lung tissue injury under oxidative stress [105].

### 5.3. Molecular Events by Radon

Radon exposure interferes with multiple molecular processes, including genetic integrity, protein regulation, and intracellular signaling. At the DNA level, radiation-induced damage leads to mutations and chromosomal abnormalities that drive genomic instability. Protein function is altered through changes in expression and post-translational modifications, disrupting stress responses and repair mechanisms. Key signaling pathways, such as those governing apoptosis, cell growth, and antioxidant defense, are also affected. These molecular events do not operate in isolation; rather, they interact to create a cellular environment prone to transformation. Understanding how radon simultaneously perturbs DNA, protein networks, and signaling cascades offers critical insight into its carcinogenic potential and may guide future preventive or therapeutic strategies.

#### 5.3.1. DNA and Chromosome

Radon exposure causes direct DNA damage and chromosomal instability, undermining genomic integrity. The α-particles released from radon decay can induce gene mutations, structural chromosome abnormalities, and widespread disruption of the genetic code. These alterations compromise the fidelity of cellular replication and lay the groundwork for malignant transformation. The persistent nature of this damage suggests that genomic instability may serve not only as a consequence of exposure, but also as a driving force in radon-induced tumorigenesis.

(1) Genetic mutation

As a source of high-linear energy transfer (LET) ionizing radiation, radon induces genetic mutations primarily through direct α-particle-induced DNA damage. Affected genes include tumor suppressors and DNA repair regulators such as *TP53*, *HPRT*, *TK*, and *EGFR*, with mutations frequently occurring as gene deletions rather than large fragment breaks. The loss of these critical genes disrupts cell cycle control and promotes unregulated proliferation, contributing to tumorigenesis [134].

Mutations in *TP53*, located on chromosome 17, are among the most studied. This gene encodes the p53 protein, which governs DNA repair, apoptosis, and cell cycle arrest. Mutation frequencies reach approximately 26% in miners and 24% in individuals living in high-radon environments [135]. These mutations are predominantly missense, clustered in exons 5–8, which encode the DNA-binding domain [136]. Such alterations impair p53’s tumor-suppressive function and may confer oncogenic properties. Both histological and molecular studies have confirmed *TP53* mutations in radon-related lung cancers, particularly large cell and squamous cell carcinomas [137,138].

The *HPRT* gene, located on the X chromosome, is also vulnerable. Elevated mutation rates have been observed in exposed individuals, often correlating with micronucleus formation and showing a linear dose–response relationship [78,139]. About half of these mutations involve full gene deletions, while others involve rearrangements [140]. Comparisons with X-ray exposure indicate similar mutation numbers but different patterns of genomic disruption [141].

Similarly, the *TK* gene, essential for DNA synthesis and repair, exhibits high mutation frequencies under radon exposure. In TK6 lymphocytes, approximately 82% of radon-induced mutations are full deletions, compared to 74% under X-rays [78,142]. This suggests that α-particles may exert greater mutagenic potential on this gene [143,144].

Mutations in *KRAS* and *EGFR* have also been associated with radon exposure, particularly in non-smoking lung cancer patients, highlighting radon’s potential role in specific cancer subtypes [145,146]. These findings collectively suggest that radon acts as a potent mutagen, targeting key genomic regions involved in cell regulation. The accumulation of such mutations may be a central mechanism by which chronic radon exposure promotes lung carcinogenesis.

(2) Chromosomal aberration

Chromosomal aberrations, including structural changes and numerical variations, are key indicators of ionizing radiation exposure. Radon and its decay products cause chromosomal damage, detectable through cytogenetic assays like multicolor fluorescence in situ hybridization (mFISH) and micronucleus testing, which assess radiation-induced genomic instability [147].

Stable chromosomal aberrations are long-lasting biomarkers of radon exposure [148]. Evidence suggests that radon can alter both chromosome structure and number, thereby contributing to its carcinogenic potential [149]. Comparative studies show that the relative biological effectiveness (RBE) of radon-induced aberrations, when compared with γ-rays, ranges from 16 to 38, highlighting a pronounced dose-rate dependency and reinforcing its value in radiation risk assessment [150]. A cytogenetic survey involving 1323 individuals reported a significantly higher frequency of chromosomal aberrations among 225 occupationally exposed workers [151].

Children living in high-radon environments show a significant increase in chromosomal aberrations in their peripheral blood, confirming its genotoxic effects, compared to those in low-radon areas [152]. In a genetic epidemiological study, specific single-nucleotide polymorphisms (SNPs) in DNA repair genes (such as rs13181, rs17655 and rs1136410) were associated with increased chromosomal aberration frequency in radon-exposed children, suggesting potential radiosensitivity markers [153]. In Slovenia, an elementary school with high indoor radon concentrations reported increased cytogenetic damage in students. The incidence of structural chromosomal aberrations ranged from 0.5% to 4% in the exposed group, compared to 0.5% to 2.5% in controls. Micronucleus counts averaged 6.52 per 500 cells in exposed students, with some reaching 15, whereas controls showed a maximum of 9 [154].

However, the relationship between chromosomal aberration rates and radon dose is not linear. Increased chromosomal translocations were observed in peripheral blood lymphocytes exposed to radon, but aberration rates declined at very high exposure levels (>3000 WLM), suggesting distinct cellular responses at extreme doses [78]. In vitro studies demonstrated a linear dose-dependent increase in chromosomal aberrations above 10 mGy. However, at lower doses, aberration frequencies did not follow a simple extrapolation, with a minimum observed at approximately 0.03 mGy, possibly due to environmental radiation background and residual effects of the Chernobyl nuclear accident [155].

Radon-induced chromosomal deletions are not randomly distributed but localized in specific regions. Studies have identified frequent deletions on chromosome 15q in radon-exposed rats, which are similar to deletions observed in human lung cancer, suggesting its involvement in tumorigenesis [134,156]. Additionally, genome-wide association studies linked deletions in chromosome 5p15.33 to increased lung cancer risk from radon exposure [146]. Human bronchial epithelial cells exposed to radon showed chromosomal losses and rearrangements, particularly affecting chromosome 8p21.3-p22 and chromosome 14q12-q13. Structural rearrangements such as t(12;13) and t(4;10) were also noted [157]. Further studies confirmed deletions in chromosome 8p21.3–p22, a region containing tumor suppressor genes, while deletions in chromosome 14 were mapped to a 0.5–1.7 cM range, potentially affecting multiple tumor suppressor genes. Rearrangements near the D14S306 locus on chromosome 14 may further contribute to gene function loss [134].

However, some studies do not support a strong correlation between radon exposure and chromosomal aberrations. One study found that prolonged exposure to high indoor radon levels did not increase stable or unstable chromosomal aberrations in peripheral blood lymphocytes detected by FISH chromosome painting [158]. Another study in a high-radon area (Takandeang Village) reveals no significant differences in chromosomal aberration or micronucleus frequencies compared to a control group (Topoyo Village), suggesting other factors, such as aging, may contribute to observed chromosomal changes [159]. These findings indicate that the relationship between radon exposure and chromosomal aberrations may be more complex than initially assumed.

(3) Genomic instability

Radiation-induced genomic instability (RIGI) is a hallmark of delayed radiation damage, characterized by elevated rates of chromosomal abnormalities, gene mutations, amplifications, and cell death across multiple cell generations [160,161]. This phenomenon is typically detected by observing delayed chromosomal changes, which serve as important markers for potential tumorigenesis [162].

Radon-derived α-particles can induce persistent chromosomal damage that extends beyond immediate deletions, leading to stable cytogenetic abnormalities that are propagated through successive divisions [78]. In human bronchial epithelial cells, fractionated α-irradiation has been shown to promote long-term genomic instability, including increased binucleated cells, micronuclei, and altered cell phenotypes, which are early indicators of malignant transformation [163]. Similar instability has been observed in hematopoietic stem/progenitor cells, where α-radiation leads to complex chromosomal aberrations in T-cells and novel rearrangements in their descendants [164]. These alterations can accumulate, increasing the risk of transformation into cancerous cells [53]. Notably, *TP53* mutations, which are commonly observed in thorotrast-associated cancers, may result from sustained genomic instability [165].

Individual susceptibility also modulates RIGI. Genetic variations in DNA repair and oxidative stress response proteins influence cellular sensitivity to radiation [166]. For instance, *hOGG1* polymorphisms impact the efficiency of oxidative DNA damage repair following radon exposure [167]. Likewise, *Brca1*, a key gene in clustered DNA damage repair, plays a protective role; its deficiency exacerbates chromosomal instability, while partial expression enhances repair fidelity [168].

Nevertheless, evidence on the universality of RIGI is mixed. Studies from high-background radiation areas such as Ramsar, Iran, found no significant difference in leukocyte telomere length between exposed individuals and controls [169]. Similarly, bone marrow cells from mice exposed to ^224^Ra did not exhibit transmissible chromosomal instability within 100 days, though instability could be induced under in vitro conditions or after transplantation [170].

Cells deploy four main pathways to repair double-strand DNA breaks: homologous recombination, classical and alternative non-homologous end joining, and single-strand annealing [171]. The balance among these mechanisms affects genomic integrity, influencing both survival and carcinogenic potential under radon exposure. Understanding their regulation is essential for mitigating radon-associated health risks and guiding therapeutic uses of high-LET radiation.

(4) Epigenetic modifications

Radon exposure induces epigenetic alterations, particularly in DNA methylation, which are implicated in tumorigenesis, immune function, and neurological disorders. Long-term high-dose exposure can lead to genome-wide methylation changes. Specific methylation differences have been observed in *TIMP2*, *EMP2*, *CPT1B*, *AMD1*, and *SLC43A2*, genes involved in processes such as cell adhesion and polarity in lung cancer [172]. Radon is also associated with hypermethylation of tumor suppressor gene promoters like *CDKN2A* and *MGMT*, potentially leading to gene silencing and cancer development [128].

In miners, 191 differentially methylated sites have been identified compared to surface workers, involving genes linked to neurotransmission, immunity, and cell adhesion, suggesting biological impacts on lung tissue [173]. Similarly, plutonium-related lung adenocarcinoma shows promoter hypermethylation of *p16*, a key cell cycle regulator, with a clear dose–response pattern [100].

In the general population, residential radon exposure correlates with methylation changes at specific CpG sites, particularly in pregnant women and children [174]. Notably, single high-dose exposure tends to reduce methylation, while low-dose fractionated radiation more often increases it. These patterns may serve as markers for early detection and intervention in α-particle-related diseases [175].

(5) Differential expression genes

Radon exposure induces DNA damage through α-particles, leading to altered RNA expression and downstream disruptions in cell growth, differentiation, and apoptosis [77]. Human and animal studies have confirmed extensive gene expression changes following radon exposure.

In miners, differential display analysis identified 30 cDNA fragments with altered expression between lung tumors and adjacent normal tissue. Six fragments were sequenced, including five (CG2, CG7, CG8, CA5, and CC6) with low homology to known GenBank entries, suggesting novel sequences. One fragment, CG3, showed 95% homology with the ribosomal protein L27a gene [176]. In vitro experiments with human lung fibroblasts revealed 208 radiation-responsive genes after α-particle exposure; 32% were upregulated, and 68% downregulated. Notably, *GDF15*, which is a marker of lung injury, was upregulated threefold and is involved in cell cycle regulation, chromosomal instability, and differentiation, indicating a potential role in radon-induced carcinogenesis [177].

Animal studies further confirmed altered expression in blood and respiratory epithelial cells. Several known genes were linked to cell proliferation, oxidative stress, apoptosis, and tumor progression. Upregulated genes include *E-cadherin*, *PTK*, and *HnRNP K*, while downregulated genes such as four-transmembrane protein and ribosomal protein L19 are involved in tumor suppression and apoptosis regulation [178]. Radon exposure also increased *E-cadherin* expression and suppressed *RPA1* and *CK1δ* in mouse bone marrow cells [179].

Interestingly, low-dose exposure may have therapeutic effects. In MCF-7 breast cancer cells, α-particles modulated apoptosis-related genes: *Bax* and *Bcl-2* were downregulated, while *Bcl-xL* and *Bcl-xS* were upregulated, potentially enhancing sensitivity to apoptosis and improving chemotherapy response [107].

#### 5.3.2. Non-Coding RNAs

Non-coding RNAs (ncRNAs), including microRNAs (miRNAs), long non-coding RNAs (lncRNAs), and circular RNAs (circRNAs), regulate gene expression through diverse mechanisms such as mRNA degradation, translational repression, chromatin remodeling, and DNA methylation. They are involved in critical cellular processes including development, apoptosis, and tumorigenesis, and their dysregulation has been linked to various diseases [180]. Increasing evidence suggests that radon exposure alters ncRNA expression, contributing to radiation-induced cellular dysfunction.

Several miRNAs are differentially expressed following radon exposure. In human bronchial epithelial cells, miR-16, miR-15, miR-23, miR-19, and miR-125 were upregulated, whereas let-7, miR-194, miR-373, miR-124, miR-146, miR-369, and miR-652 were downregulated, potentially affecting proliferation, apoptosis, and DNA methylation [103]. Reduced let-7 levels and increased *K-ras* expression were observed both in vitro and in vivo [181]. In mouse skin, long-term radon inhalation induced tissue damage and altered 57 miRNAs, including miR-206-3p and miR-378a-3p, which target fatty acid synthase (FASN) and may contribute to cutaneous toxicity [84].

LncRNAs are also sensitive to radon. One study identified 1256 differentially expressed lncRNA transcripts in mouse lung tissue after radon exposure, implicating pathways such as ErbB and Notch in pulmonary injury. Increased expression of *K-ras* and *ErbB2* further supports their involvement in radon-induced toxicity [182]. While the precise mechanisms remain unclear, lncRNAs may modulate gene expression by affecting histone modifications and chromatin structure.

Additionally, circRNAs, known to regulate miRNAs and proteins, were differentially expressed in radon-exposed mouse lungs. Their dysregulation was associated with inflammation and elevated Caspase-3 levels, suggesting a role in lung injury. However, their contribution to lung cancer remains uncertain and requires further investigation [83].

#### 5.3.3. Proteins Alterations

Radon exposure induces significant changes in protein expression, affecting cellular processes such as oxidative stress response, DNA repair, and inflammation. Proteomic analyses in lung, serum, and skin tissues have identified numerous differentially expressed proteins, offering potential biomarkers for radon-induced injury and disease progression.

In lung tissue, both animal and human studies have demonstrated consistent alterations in protein levels following radon exposure. Comparative proteomics revealed the upregulation of RAGE and S100A6 in rat lungs and human bronchial epithelial cells, suggesting their potential as markers of radon-induced lung damage [183]. Additional serum markers identified among uranium miners include APOC1–3, ORM1–2, ANTXR1, GCN1, and CIP2A. The iTRAQ and ELISA methods confirmed the differential expression of GCN1 and CIP2A in underground workers, indicating their value for early detection [184]. In miners with radon-related squamous cell carcinoma, upregulated levels of K1, K10, CFH, and fragmented haptoglobin were reported. Notably, CYFRA 21-1 showed high diagnostic sensitivity and specificity [185].

Co-exposure to radon and cigarette smoke also altered protein expression in rat lungs. Downregulated proteins included CcO, Serpin B11, DDAH1, and MLP4, while upregulated proteins included ALDH, RAGE, Trx, HSP27, HSP70, and S100A6, reinforcing their association with lung injury [186]. In high-radon areas, serum proteomics from non-smokers revealed 799 differentially expressed proteins. Among these, 25 proteins, including IGHA2, TRIM43, BLINK, KIR3DL1, and ZNF469, were detected only in lung cancer patients or exposed individuals but not in unexposed healthy controls. These proteins may help identify populations at risk [187].

In addition to pulmonary effects, radon exposure alters skin protein profiles. In mice, proteins involved in lipid metabolism (THRSP, ACACA, FASN, ACLY) were upregulated, while Mup17, S100a9, and Mup1 were downregulated. FASN, in particular, may contribute to radon-induced skin injury [84]. These findings highlight the tissue-specific proteomic responses to radon and provide a foundation for biomarker development.

#### 5.3.4. Intracellular Signaling Pathway

Radon exposure activates multiple intracellular signaling pathways, notably p53, PI3K/AKT/mTOR, and Nrf2/ARE, which mediate responses to oxidative stress, cell proliferation, and survival.

The p53 pathway is a central regulator of DNA repair and apoptosis. Radon disrupts this pathway, potentially impairing mitochondrial function and promoting malignant transformation in lung tissue [98]. While activation of p53 can serve as a protective response to DNA damage, persistent dysregulation may contribute to carcinogenesis. Thus, modulation of p53-related pathways presents a possible strategy for preventing or treating radon-induced pulmonary diseases [77]. The Nrf2/ARE pathway plays a key role in regulating antioxidant defense. In BEAS-2B cells, radon exposure alters the expression of Nrf2 and its downstream enzymes, indicating that activation of this pathway may offer limited protection against oxidative stress [105].

Overall, radon-induced changes in signaling pathways affect oxidative stress regulation, energy metabolism, and cellular homeostasis. These molecular disruptions contribute to increased cancer risk and underscore the complex cellular mechanisms underlying radon-related diseases.

### 5.4. Special Radiation Effects

Beyond direct cellular damage, radon exposure exerts its effects through several unique radiation phenomena that amplify biological responses. The bystander effect (BE) enables non-irradiated cells to exhibit radiation-like damage via signals from nearby irradiated cells, expanding the overall impact of radon. The enhanced deposition effect (EDE) leads to uneven alpha particle distribution, increasing localized lung tissue doses and intensifying injury. Additionally, the inverse dose-rate effect indicates that low-dose, long-term exposure may cause more harm than short-term high doses due to insufficient DNA repair. These effects complicate the biological consequences of radon exposure, highlighting the need to consider non-targeted and dose-rate-related mechanisms when evaluating its health risks.

#### 5.4.1. Bystander Effect (BE)

The bystander effect refers to biological responses in non-irradiated cells induced by signals from neighboring irradiated cells. This effect is particularly evident in radon exposure and plays a significant role at low doses, where it contributes to nonlinear dose–response relationships and the inverse dose-rate effect [188].

Irradiated cells release ROS, cytokines, growth factors, and metabolic byproducts that affect surrounding cells through gap junctions or extracellular signaling [78,189,190]. These signals can lead to DNA double-strand breaks (γ-H2AX), chromosomal aberrations, changes in apoptosis and cell cycle, and altered gene expression [78,190]. At low-dose α-particle radiation, where direct hits may be insufficient for damage, BE dominates cellular responses. Studies show that one irradiated lung epithelial cell can affect up to 50 neighboring cells [191]. This leads to a downward-curving dose–response relationship and strengthens the inverse dose-rate effect [191,192].

Models of radon exposure suggest a combined effect of direct α-particle damage and BE on lung tissue, which complicates risk assessment in residential and occupational environments [193]. In BEAS-2B cells, conditioned medium from irradiated cells induces increased ROS, reduced p53 and *CDKN1A* expression, and proliferation in non-irradiated cells [194]. The bystander effect also triggers DNA damage responses, apoptosis, or autophagy, depending on cellular context [195,196]. These interconnected pathways highlight the importance of BE in radon-induced carcinogenesis, particularly under chronic low-dose exposure [196].

#### 5.4.2. Enhanced Deposition Effect (EDE)

The enhanced deposition effect describes the increased accumulation of radon and its progeny in the respiratory tract influenced by factors such as humidity, airflow, and aerosol properties. At high radon concentrations, EDE causes more radon progeny to deposit in the nasal and oral airways, reducing the amount reaching the lungs [197,198].

Humidity, airflow speed and direction, aerosol particle size, and charge all affect EDE. For example, at radon levels above 50 kBq/m^3^ and around 52% relative humidity, radon progeny like ^218^Po show reduced particle sizes (~0.40 ± 0.02 nm), enhancing deposition in upper airways and decreasing lung dose [197,198,199,200]. Increased airflow can push particles deeper into the lungs, increasing deposition in lower regions.

High radon concentrations can alter the arithmetic mean diameter (AMD) of radon progeny particles through changes in particle neutralization and aerosol conditions, with the magnitude of this effect also depending on environmental factors such as relative humidity. Under the specific ventilation, relative humidity, aerosol particle-size, and chamber conditions considered in the cited study, the lung dose per unit radon concentration was modeled to decrease to approximately one-quarter of the reference value [199]. This finding is condition-dependent and should not be interpreted as indicating that each unit increase in radon concentration causes a proportional decrease in lung dose.

EDE results in uneven distribution of radon decay products in the lungs, concentrating doses in certain regions and thereby increasing cancer risk. This heterogeneous deposition relates closely to the inverse dose-rate effect (IDRE) and is critical for accurate radon risk assessment. Breathing patterns and lung anatomy influence this uneven dose distribution, emphasizing the importance of EDE in lung cancer risk modeling [85,89,198,200].

#### 5.4.3. Inverse Dose-Rate Effect (IDRE)

The inverse dose-rate effect (IDRE) refers to the observation that, for the same cumulative radon progeny exposure, the lung cancer risk per unit exposure may be greater when the exposure is delivered at a lower exposure rate over a longer duration than at a higher exposure rate over a shorter duration [54]. IDRE has primarily been described in occupational and experimental settings, particularly at relatively high cumulative radon exposures. Radon concentration, exposure rate, cumulative exposure, and exposure duration should therefore be considered as distinct exposure metrics rather than used interchangeably. Some miner and experimental studies have reported variations in lung cancer risk according to exposure rate; however, these observations should not be interpreted as evidence of a protective effect of low residential radon exposure. Major pooled residential epidemiological analyses indicate an approximately linear increase in lung cancer risk with increasing radon concentration and do not support a protective threshold [49].

Mechanistically, IDRE is linked to multiple α-particle traversals of cell nuclei, replenishment of hypersensitive cell subpopulations, and the enhanced deposition effect (EDE). Due to their high mass and charge, α-particles can cause repeated nuclear hits during prolonged exposure, amplifying biological damage [54]. Low-dose, long-term exposures may allow greater opportunity for cellular repair and repopulation, whereas higher dose rates may increase cell killing and alter the population of susceptible cells [191]. EDE influences IDRE by altering radon progeny deposition, reducing lung dose rates despite higher radon levels, which contributes to the IDRE phenomenon [199,200]. The reduced lung dose from EDE, rather than a simple decline in progeny-induced damage, plays a key role in IDRE [199].

In miners with relatively high cumulative exposure, an inverse dose-rate pattern has been observed in which, for the same cumulative exposure, the lung cancer risk per unit exposure tends to be greater at lower exposure rates and longer exposure durations. At lower cumulative exposures, the inverse dose-rate effect appears to diminish, and its relevance to typical residential exposure conditions remains uncertain [54].

Particle size reduction at high radon concentrations, especially with higher humidity, also lowers lung dose rate per radon unit, providing another explanation for IDRE [199]. The bystander effect and IDRE share underlying mechanisms; IDRE may result from replenishment of bystander-sensitive subpopulations during exposure [196].

The magnitude of IDRE may vary according to cumulative exposure, exposure rate, and exposure duration. However, exposure duration should be interpreted together with exposure rate and cumulative exposure rather than as an independent determinant of risk. At cumulative exposures below approximately 50 WLM, the inverse dose-rate effect has been reported to diminish [54,201]. Genetic background, health, and lifestyle also modulate radon’s biological effects under similar exposures [202].

IDRE should be interpreted as an effect modification by exposure rate under specified cumulative-exposure conditions rather than as evidence against a linear relationship between cumulative radon exposure and lung cancer risk. Findings from miner studies should therefore be extrapolated to low-level residential exposure with caution [202]. At very low cumulative exposures, the probability of multiple α-particle traversals of the same target cell is reduced, which may weaken exposure-rate-dependent effects. Studies on smoking and non-smoking uranium miners confirm radon as a lung cancer risk factor. Rat experiments found radon-induced chromosomal aberrations 13 times higher than comparable doses of ^60^Co, illustrating that IDRE mechanisms may differ between residential and mining environments [203]. These findings may contribute to a better understanding of radon carcinogenesis and the relevance of dose-rate effects to low-level residential exposure.

### 5.5. Effects of Radon on Gut Microbiota

Radon exposure disrupts the gut microbiota by inducing dysbiosis, reducing microbial metabolic activity, and causing adaptive changes in specific microbial populations. These alterations collectively affect gut microbial structure and function.

Environmental and in vitro studies demonstrate that radon significantly suppresses microbial metabolic activity. In a radon-contaminated laboratory, soil microbes exposed to varying radon levels showed markedly decreased metabolic activity, likely due to the damaging effects of ionizing radiation [204]. Similarly, bacterial cultures exposed to radon exhibited reduced oxygen consumption compared to controls, indicating inhibited bacterial growth and metabolism [205]. In vivo studies have shown that radon exposure alters the abundance of beneficial gut bacteria, particularly those responsible for producing short-chain fatty acids (SCFAs). As SCFAs are essential for maintaining gut homeostasis, their reduction may lead to impaired gut function and increased susceptibility to disease [79].

Despite these adverse effects, some microbial populations display tolerance to radon. Investigations of extremophilic bacteria from radon-rich environments, such as radon springs, have identified species, especially within the Actinobacteria phylum, that exhibit strong resistance to radiation and oxidative stress. Lipidomics analyses suggest that these bacteria adapt by modulating fatty acid content and membrane composition, highlighting potential survival strategies under radon exposure [206].

## 6. Therapeutic Effects of Radon

Radon has a long history of therapeutic use, particularly in Central Europe, where radon-containing springs, galleries, and spa facilities have been used in the management of chronic inflammatory and musculoskeletal disorders. The therapeutic use of radon emerged in the early 20th century after the discovery of radon and the recognition of radioactivity in several mineral springs, and it subsequently became incorporated into spa-based treatments in countries such as Germany and Austria [207]. In spa-based therapeutic settings, radon exposure has been administered through inhalation in galleries or mines, bathing in radon-containing water, or other spa-based approaches, with the absorbed dose remaining relatively low compared with therapeutic radiation used in oncology [208].

Current clinical evidence is concentrated mainly on rheumatic and musculoskeletal disorders, including ankylosing spondylitis, rheumatoid arthritis, osteoarthritis, and chronic back pain. Controlled trials and meta-analyses have reported reductions in pain and, in some studies, prolonged symptomatic improvement after radon-containing spa therapy, although differences in treatment protocols, concomitant spa interventions, sample sizes, and study quality limit the strength of these conclusions [14]. In studies involving multimodal spa therapy, the independent contribution of radon cannot always be distinguished from the effects of thermal exposure, exercise, balneotherapy, or other concomitant treatments. Radon-enriched inhalation therapy has also been investigated for inflammatory respiratory conditions such as allergic rhinitis, chronic rhinosinusitis, and asthma, with several small prospective studies reporting improvements in objective measures of nasal or pulmonary function [209].

Beyond these indications, experimental and clinical studies have explored possible effects of radon exposure on immune regulation, oxidative stress, and inflammatory signaling, providing potential mechanistic explanations for some reported therapeutic responses [208]. However, evidence for broader applications outside rheumatic and selected inflammatory disorders remains limited, and many reported benefits are based on small studies, combined spa interventions, or observational designs. Therefore, the therapeutic potential of radon should be interpreted cautiously, and further well-designed randomized controlled trials are needed to establish efficacy, optimal exposure conditions, and long-term safety. Despite its historical and ongoing use in selected spa settings, radon therapy has not been widely incorporated into mainstream clinical practice, and its efficacy remains insufficiently established for many proposed indications.

**Figure 5:**
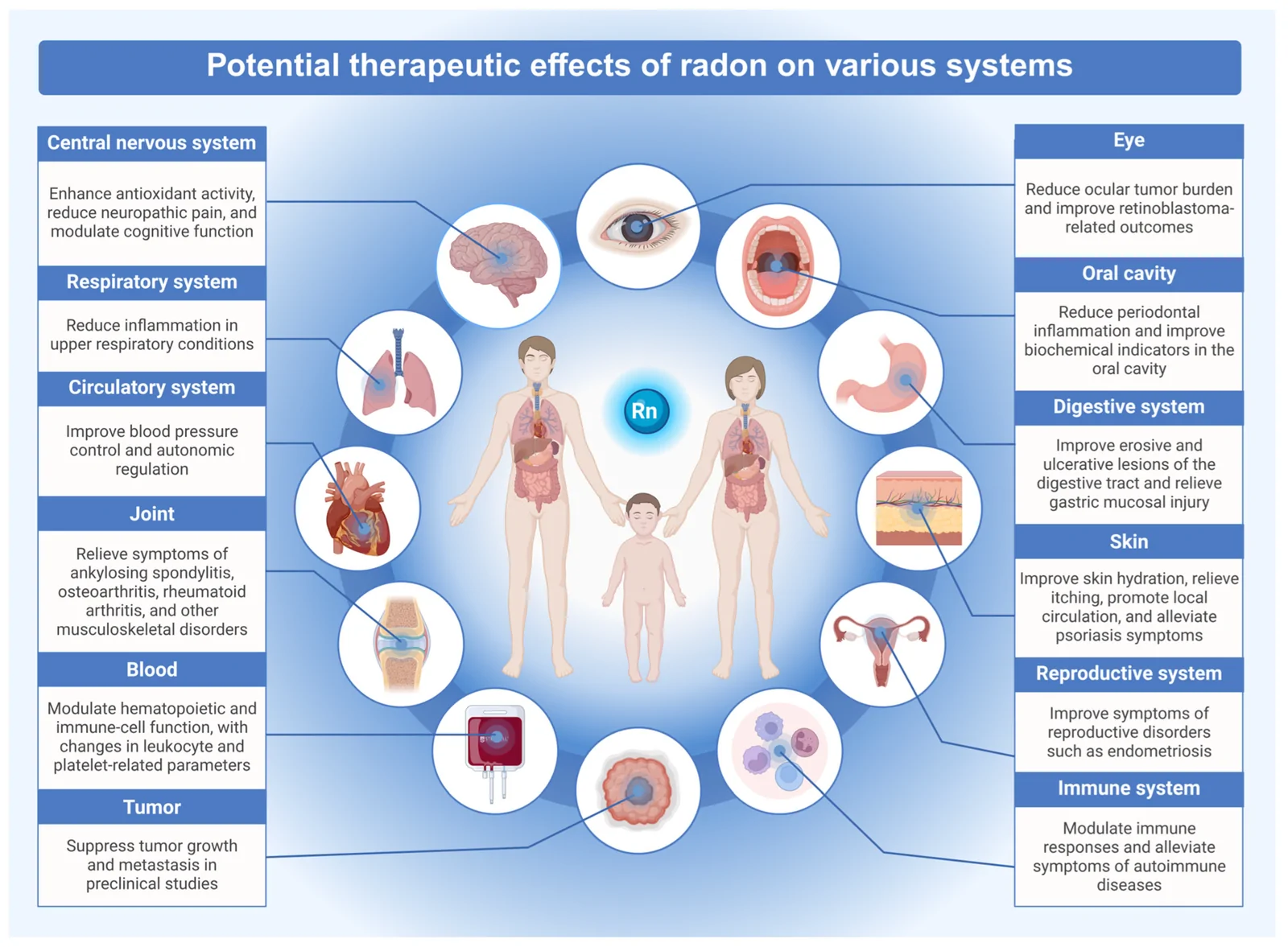
Potential therapeutic effects of radon on various systems.

**Figure 6:**
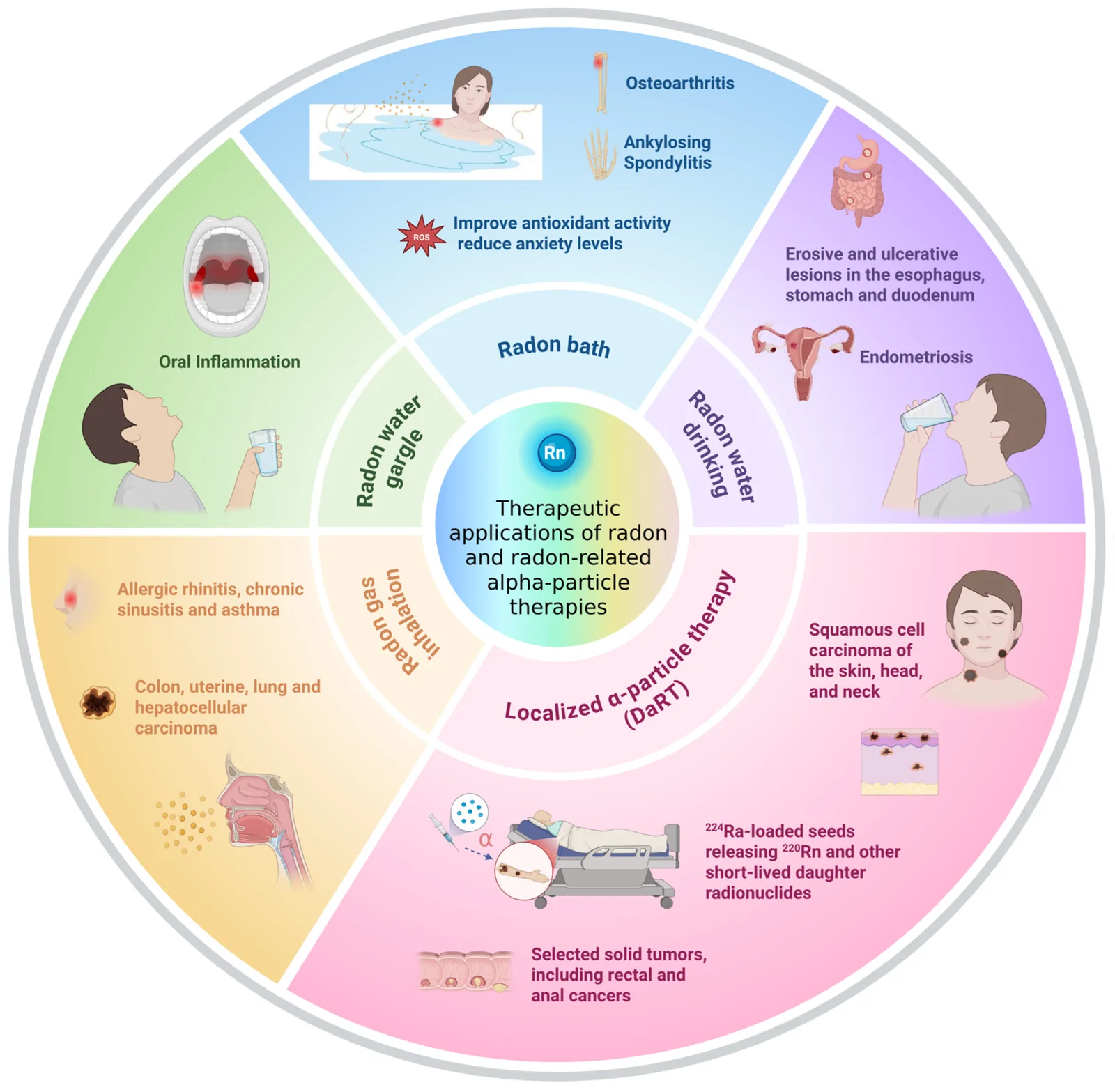
Therapeutic applications of radon and radon-related alpha-particle therapies.

Clinical and experimental studies evaluating the therapeutic applications of radon are summarized in Table 1, while the reported organ- and disease-related therapeutic effects are illustrated in Figure 5. In addition, Figure 6 presents selected therapeutic approaches involving radon or radon-related alpha-emitting radionuclides. Importantly, conventional radon spa therapy should be distinguished from modern localized alpha-particle treatments such as Diffusing Alpha-emitters Radiation Therapy (DaRT), which uses ^224^Ra-loaded sources that release short-lived daughter radionuclides, including ^220^Rn, within the tumor microenvironment. DaRT therefore represents a distinct form of localized alpha therapy rather than conventional radon inhalation or balneotherapy.

### 6.1. Rheumatic Diseases

Radon-containing spa therapy has been investigated for the management of several rheumatic diseases. In ankylosing spondylitis (AS), natural radon-rich water and steam from the Gastein Valley in Austria have been used therapeutically for over half a century. Herold et al. conducted randomized controlled trials involving hundreds of AS patients, reporting longer-term symptomatic improvement following hydrotherapy combined with radon, although the independent contribution of radon cannot always be distinguished from other components of spa therapy, with effects sustained for up to 40 weeks [14]. In a cohort of 83 AS patients, a 3–4-week course of radon therapy in Badgastein resulted in elevated TGF-β1 levels, particularly in those who experienced a reduction in morning pain [14]. Other studies reported improvements in quality-of-life measures following low-dose radon spa therapy, with some effects persisting for 6–9 months [14]. These findings suggest that radon spa therapy may provide adjunctive symptomatic benefits in patients with AS, although further high-quality controlled studies are required to establish its clinical efficacy. More recently, a 2026 register-based study of 257 patients with ankylosing spondyloarthritis reported improvements in disease activity and functional performance following multimodal spa rehabilitation that included low-dose radon; however, because radon was administered as part of a comprehensive rehabilitation program, its independent contribution could not be determined [253].

Radon-containing interventions have also been investigated in osteoporosis and osteochondrosis. In individuals at risk of age-related osteoporosis, radon bath therapy combined with exercise improved bone metabolism regulators and reduced physical discomfort [214]. In patients with osteochondrosis and nerve root syndrome, radon water treatment reduced pain and enhanced spinal mobility [14]. A 21-day multidisciplinary spa program in Przerzeczyn-Zdrój, Poland, which included radon-sulphide water, physical therapy, and psychological support, showed superior pain relief compared to outpatient treatment alone [14]. These results highlight the potential of radon baths as part of a comprehensive approach to bone and joint disorders.

Radon-containing interventions have also been investigated in knee osteoarthritis (OA), with several studies reporting improvements in pain and function. A long-term study combining radon baths and extracorporeal shock-wave therapy in stage II–III knee OA patients demonstrated significant reductions in pain, increased joint mobility, and improved quality of life. Positive effects persisted for up to 12 months, and 82% of patients achieved stable remission [14]. A systematic review of spa therapies across Europe and the Middle East reported improvements in OA symptoms, including reduced pain and better function [14]. Further research showed that combined cryotherapy and radon therapy positively affected the neuroendocrine and immune systems, reducing inflammation and supporting individualized regenerative treatments [14]. Cytogenetic improvements were also observed; patients receiving radon baths exhibited a marked decrease in trisomy 7-positive synovial cells, correlating with pain relief [14]. A 2-week trial at the Bad Gastein radon gallery showed sustained reductions in self-reported pain among elderly knee OA patients [14]. Moreover, a randomized multicenter trial reported longer-term reductions in pain following radon-containing spa therapy, with differences observed for several months after treatment [14]. A subsequent review concluded that when used as part of multifactorial treatment, radon therapy provides clinically relevant improvements in pain and function in knee OA [254].

Radon-containing spa therapy has also been investigated in patients with rheumatoid arthritis (RA). In a study involving 50 RA patients receiving 21-day radon spa therapy in the Czech Republic, significant improvements were observed in quality-of-life parameters including pain, vitality, and emotional well-being [14]. Another trial comparing natural radon–carbon dioxide baths with artificial CO_2_ baths found similar short-term effects, but only the radon group exhibited sustained improvement at 6 months [14]. A double-blind study further confirmed that radon–carbon dioxide baths reduced steroid and NSAID use more effectively than control treatments, indicating long-term benefit [14].

In osteoporosis, low-dose radon included in multimodal spa therapy demonstrated both immediate and lasting effects on bone metabolism and discomfort reduction. A meta-analysis of 338 patients found that while immediate outcomes were comparable to controls, radon therapy yielded superior pain relief at 3 and 6 months post-treatment in conditions such as degenerative spinal disease, RA, and AS [14].

However, some studies reported limited additional benefit. One trial indicated that radon treatments and carbon dioxide injections offered no clear advantage over standard multifactorial spa care [255]. Another study comparing radon thermal baths with warm water baths in 116 patients with musculoskeletal disorders showed that radon led to more rapid pain reduction and improvement in morning stiffness and pressure pain threshold. Nevertheless, long-term follow-up revealed similar pain outcomes in both groups at 12 and 24 weeks [256]. These findings suggest radon therapy may offer superior short-term relief, while long-term effects may be comparable to conventional thermal treatments.

### 6.2. Digestive System

Radon-containing interventions have been investigated in selected digestive tract conditions. In patients with recurrent erosive and ulcerative lesions of the esophagus, stomach, and duodenum, a treatment regimen combining radon baths, mineral water ingestion, and conventional antiulcer drugs resulted in significant clinical improvement. Clinical improvement was reported following this multimodal intervention; however, the independent contribution of radon cannot be determined because radon baths, mineral water, and conventional antiulcer treatment were administered together [14].

Experimental studies further support the gastrointestinal protective effects of radon. In a mouse model of ethanol-induced gastric mucosal injury, pretreatment with radon or hot spring water significantly reduced lipid peroxide levels in the stomach, suggesting a protective antioxidative effect [14].

Epidemiological data also indicate beneficial effects of radon hot spring bathing on gastrointestinal health. A cross-sectional study conducted in Misasa, Japan, involving 5250 adults, found a significant association between frequent radon hot spring bathing (≥1 time/week) and reduced symptoms of gastroenteritis. Additionally, frequent bathing was positively correlated with improved self-rated health compared to non-bathing individuals [227].

Together, these findings suggest that radon-containing spa interventions warrant further investigation in gastrointestinal conditions; however, current evidence does not establish an independent therapeutic effect of radon.

### 6.3. Central Nervous System

Radon has demonstrated neuroprotective potential through the modulation of oxidative stress and antioxidant activity. Inhalation of radon has been shown to enhance the activity of antioxidant enzymes, particularly superoxide dismutase, and to confer protection against transient global cerebral ischemic injury in gerbils [14]. Similarly, low-dose X-irradiation (0.5 Gy) reduced oxidative stress and immobility behavior in a mouse forced swim test, supporting its antioxidative effect on the brain [14].

Radon may also play a role in pain modulation. In a mouse model of chronic constriction injury (CCI), combined treatment with radon inhalation (1000 Bq/m^3^ for 24 h) and pregabalin produced greater analgesic effects than either intervention alone. This additive effect is thought to be mediated by enhanced antioxidative function [229].

In addition to its antioxidative and analgesic properties, radon exposure influences neural metabolism. Metabolomic analysis revealed that radon alters sulfur-related metabolites in the brain, including increased L-methionine levels and decreased concentrations of urea, glutathione (G-SH), and sulfite ions. Despite a reduction in the G-SH/GS-SG ratio, levels of glutathione monosulfide (G-S-SH) and cysteine monosulfide (Cys-S-SH) increased, indicating a shift in redox homeostasis following radon exposure [14].

Radon hormesis balneotherapy, particularly when combined with mineral-rich thermal waters such as those from Tskaltubo springs, has also shown beneficial effects on behavioral and biochemical markers. In animal studies, this combined treatment enhanced antioxidant capacity, improved the G-SH/GS-SG ratio in the brain, and alleviated anxiety-like behaviors [14].

Collectively, preclinical findings indicate that radon exposure can modify oxidative, metabolic, and behavioral endpoints in experimental models; whether these effects translate into clinical neurological benefit remains uncertain.

### 6.4. Respiratory System

Radon inhalation has been investigated for its potential anti-inflammatory effects in the upper respiratory tract. In a prospective study, patients with chronic sinonasal inflammation received a 14-day treatment of warm radon vapor inhalation followed by nasal aerosol at Merano Therme. Clinical improvements were reported following this intervention; however, the available evidence is insufficient to establish radon-enriched spa therapy as an alternative to standard treatment for refractory upper airway inflammation [14]. Another study compared radon-rich oligomineral water vapor (481 Bq/L radon, fluorine-rich) with mometasone furoate nasal spray and reported improvements in clinical and cytological parameters after two weeks of radon inhalation [234]. A systematic review reported improvements in objective nasal-function measures following radon-containing inhalation therapy in patients with allergic rhinitis and chronic rhinosinusitis, with some studies also reporting changes in airway obstruction in patients with asthma [14]. These findings suggest that radon-containing inhalation therapy warrants further investigation for inflammatory conditions of the upper respiratory tract, although the current evidence is insufficient to establish its clinical efficacy. Recent preclinical evidence has extended these observations to lower respiratory inflammation. In a 2026 murine model of LPS-induced pneumonia, radon inhalation was associated with improved clinical signs and lung architecture, suppression of Th1-related inflammatory responses, and promotion of anti-inflammatory macrophage activation [257]. However, another 2026 murine study using a different radon pre-inhalation and LPS protocol did not observe suppression of pulmonary MPO activity and instead reported enhanced lung inflammation, together with changes in several proteins identified by proteomic analysis [258]. These apparently divergent findings suggest that pulmonary responses to radon may depend strongly on exposure timing, concentration, duration, and the specific inflammatory model, and therefore require further experimental clarification.

Although several individual case–control and ecological studies have reported nonlinear or inverse associations between low-level radon exposure and lung cancer risk, these findings should be interpreted cautiously. Such observations are inconsistent with the larger pooled residential epidemiological analyses, which demonstrate an approximately linear increase in lung cancer risk with increasing radon concentration and provide no evidence of a protective threshold [49]. Therefore, the proposed low-dose hormetic effect on lung cancer risk should be regarded as a hypothesis requiring further investigation rather than as evidence that low-level radon exposure prevents lung cancer.

Despite these findings, controlled animal experiments have consistently demonstrated a dose-dependent increase in lung cancer incidence. In rats, lung cancer rates rose from 2.2% at 25 WLM to 77% at 1600 WLM. When combined with tobacco smoke, the carcinogenic effect was further amplified; rats exposed to 1000 WLM radon plus passive smoke exhibited a lung cancer rate of 78%, nearly double that of radon alone. Squamous cell carcinoma was the predominant histological type observed [259]. These experimental findings demonstrate carcinogenic effects at higher radon exposures but do not establish a protective effect at low residential exposure levels.

Experimental studies have reported adaptive biological responses following short-term low-dose radon exposure; however, whether such responses translate into a reduced lung cancer risk in humans remains uncertain. In one study, male Balb/c mice exposed to elevated radon levels exhibited signs of an adaptive response [14]. The possible relevance of such adaptive responses to radiation protection or therapeutic applications remains to be established.

### 6.5. Oncology and Metastasis Suppression

Possible oncological applications of radon-related exposure have been explored in preclinical studies and a limited number of clinical case reports. A report describing four cancer patients discussed radon exposure and other α-emitting radionuclides, including ^223^Ra, as experimental therapeutic approaches; however, observations from such a small uncontrolled series cannot establish clinical efficacy [14]. In preclinical studies, radon treatment has demonstrated anti-metastatic effects. In experimental mouse models, administration of radon-containing water at 68–203 Bq/L was associated with delayed progression of atopic dermatitis-like lesions in NC/Nga mice. In C57BL/6 mice inoculated with B16 melanoma cells, radon exposure before tumor inoculation was associated with fewer pulmonary metastatic foci, particularly under conditions of lower tumor burden [14]. Radon exposure was also associated with an increased IFN-γ/IL-4 ratio in splenocytes of BALB/c mice immunized with DNP-Ascaris, suggesting possible modulation of Th1-related immune responses [14].

Radon inhalation has also been described in a small case series involving patients with colon, uterine, lung, and liver cancers. The case series described changes in tumor markers and symptoms after treatment; however, the uncontrolled design and very small sample size preclude conclusions regarding therapeutic efficacy.

Case reports have also described radon-related interventions in patients who were unwilling or unable to undergo conventional chemotherapy or radiotherapy; however, such reports do not provide sufficient evidence to support radon as a primary therapeutic option. One case involved a patient with advanced prostate cancer and bone metastasis. After partial remission from chemotherapy, his PSA level rebounded to 4.8. He then received ten sessions of 0.15 Gy stimulation therapy over three weeks, followed by nightly use of a radon bed sheet for ten months. During follow-up, his PSA level decreased to 0.008, and bone metastases were no longer detected on the ^99^mTc-HMDP scan [260]. Because this observation was derived from a single case involving multiple interventions, a causal effect of radon cannot be established.

At present, the available evidence is insufficient to establish radon therapy as either an adjunctive or standalone oncological treatment. Most clinical observations are derived from case reports or very small uncontrolled studies, while much of the supporting evidence remains preclinical. Well-designed controlled clinical studies would therefore be required to determine whether radon-related interventions have reproducible therapeutic effects, as well as to evaluate appropriate exposure conditions and long-term safety.

### 6.6. Oral Diseases

Radon-containing mineral-water interventions have been investigated for their possible effects on oral inflammation and biochemical indicators. In a clinical study, participants underwent inhalation therapy and oral rinsing with radon-containing mineral water from Tskhaltubo, Georgia. Improvements in inflammatory indicators were reported following the intervention [14]; however, because the treatment involved radon-containing mineral water as a complex exposure, the independent contribution of radon cannot be determined.

Radon also influences oral biochemical activity. In a study of 132 patients aged 20–40 years with different forms of periodontitis, a 10-day intervention involving inhalation and mouth rinsing with radon-containing water was associated with lower LDH levels in oral fluid and changes in other biochemical indicators, including amylase activity and oral pH [14]. These observations suggest changes in the local biochemical environment, but they do not establish that radon itself was responsible for the reported effects.

A combined intervention involving photodynamic therapy, inhalation, and rinsing with Tskhaltubo radon-containing water has also been evaluated in patients with periodontitis. Improvements in periodontal symptoms and oral biochemical indicators were reported following treatment [14]. Because multiple interventions were administered concurrently, the independent effect of radon cannot be determined. These preliminary observations warrant further controlled investigation of radon-containing interventions in oral disease; however, current evidence is insufficient to establish a radon-specific hormetic effect or a role in disease prevention.

### 6.7. Reproductive System

Radon-containing spa interventions have been investigated as components of multimodal treatment and rehabilitation programs for endometriosis. A three-year evaluation conducted from 2014 to 2016 examined spa- and resort-based rehabilitation in patients with external genital endometriosis. A multimodal regimen combining radon-containing spa therapy, mineral-water consumption, and gonadotropin-releasing hormone agonists was associated with improved outcomes [241]. Because several interventions were administered concurrently, the independent contribution of radon cannot be determined.

Another study involved 348 women of reproductive age with stage I–III endometriosis. Participants were divided into two groups, with one receiving conventional drug therapy alone and the other receiving drug therapy combined with radon-based local treatment. The combined-treatment group showed more favorable reported outcomes than the drug-therapy-only group [242]. These findings require confirmation in rigorously designed controlled studies before a radon-specific therapeutic effect can be established.

### 6.8. Immune System

Radon-related interventions have been explored in a limited number of reports involving autoimmune diseases. One report described two patients who received radon-related therapy for 6–9 months, including one patient with pemphigus accompanied by previous myocardial infarction and diabetes mellitus and another with type I diabetes. Changes in selected disease markers and symptoms were reported during follow-up. Because immune dysregulation, including alterations in T-cell responses, contributes to the pathogenesis of several autoimmune diseases, experimental studies have explored whether low-dose radiation may influence regulatory T-cell activity and Th1/Th2-related immune responses. Such mechanistic observations, however, do not establish clinical efficacy of 222Rn therapy. Although radon-related interventions have been discussed in relation to a range of autoimmune conditions, the available evidence is largely based on case reports and experimental studies and is insufficient to establish clinical efficacy or support radon therapy as a standard treatment for refractory autoimmune diseases [261].

### 6.9. Circulatory System

Possible effects of radon-containing spa therapy on blood pressure and autonomic function have also been investigated in patients with hypertension. In a study monitoring 103 patients receiving radon spa therapy (radon concentrations of 1200 Bq/L or 600 Bq/L with 1 g/L CO_2_ at 34 °C), pain perception and radical processing capacity were assessed at three time points. Among 46 hypertensive patients, 24 h blood pressure and heart rate variability (HRV) were evaluated. The study reported sustained reductions in pain in many participants, together with changes in superoxide dismutase activity. Blood pressure also decreased during follow-up, particularly among patients receiving carbon-dioxide-containing treatment, and changes in heart rate variability were observed [262]. Because the intervention involved spa treatment with differing radon and carbon dioxide exposures, these findings do not establish an independent antihypertensive effect of radon. A recent 2026 review further proposed that radon balneotherapy may influence cardiovascular autonomic regulation through redox-sensitive and neuroimmune pathways; however, the available cardiovascular evidence remains limited, and the specific contribution of radon is difficult to separate from concomitant thermal and spa-based interventions [263].

## 7. Biological Mechanisms of Radon Therapy

Experimental and clinical studies have proposed several biological mechanisms that may contribute to responses observed following controlled radon exposure, including changes in oxidative stress, DNA damage responses, inflammatory pathways, immune regulation, and cellular adaptation. These effects have been observed across different biological systems, from molecular and cellular responses to systemic physiological changes. While low-dose radon exposure has been linked to beneficial adaptive responses, its precise mechanisms remain an area of ongoing research. This section summarizes the key biological processes underlying radon therapy, providing insights from both experimental and clinical studies.

### 7.1. Radiobiological Effects of Radon

Radon therapy is based on the radioactive properties of radon and its decay products. In radon-based spa or experimental therapeutic settings, exposure may occur through inhalation, bathing in radon-containing water, or other controlled exposure routes, depending on the intervention under investigation. As radon decays and emits α-particles, these particles interact with surrounding cells and tissues. Due to their high energy and short range, α-particles can induce highly localized ionization and damage in cellular structures, including DNA. These interactions can also lead to the ionization of water molecules and the generation of reactive oxygen species (ROS), particularly hydroxyl radicals, which may further contribute to biomolecular damage and oxidative stress [14].

Although α-particle radiation is used therapeutically in several established radionuclide-based cancer treatments, conventional radon spa or inhalation therapy should not be equated with these oncological modalities. Evidence for antitumor effects of radon exposure itself remains largely preclinical or limited to case reports. Modern localized α-particle approaches, such as DaRT, involve distinct radionuclide sources and dosimetric principles and should therefore be considered separately from conventional radon therapy. Proposed biological responses following low-dose radon exposure include changes in endogenous antioxidant systems, corticosteroid-related pathways, DNA damage responses, and immune regulation [264]; however, the extent to which these responses mediate clinically meaningful therapeutic effects remains uncertain.

### 7.2. Regulation of Oxidative Stress and Redox Balance

Radon exposure, particularly through inhalation or the use of radon-enriched hot spring water, has been shown to regulate oxidative stress and influence redox balance within the body. Several studies have explored its biological effects, highlighting its role in enhancing antioxidant activity and modulating oxidative stress responses.

#### 7.2.1. Antioxidant Enzyme Activation

Radon exposure has been shown to stimulate the activity of key antioxidant enzymes, particularly superoxide dismutase (SOD), which plays a critical role in mitigating oxidative damage. In a study mice were exposed to radon concentrations of 500 and 2000 Bq/m^3^ for 24 h, observing an increase in manganese (Mn)-SOD levels and mitochondrial SOD activity. Although the changes were not statistically significant, radon exposure was found to upregulate NF-κB, a key regulator of Mn-SOD induction, suggesting the activation of oxidative stress pathways that promote antioxidant defense [265,266].

In another study, mice exposed to radon at concentrations of 2 or 20 kBq/m^3^ for durations ranging from one to ten days exhibited decreased levels of 8-hydroxy-2′-deoxyguanosine (8-OHdG), a marker of oxidative DNA damage, in the brain and kidneys. This reduction suggests a protective effect of radon against oxidative DNA damage, potentially through enhanced Mn-SOD activity [248]. Additionally, short-term exposure to high radon concentrations (40 min per day) significantly increased SOD activity and reduced lipid peroxidation levels in mice, supporting the notion that radon exposure enhances antioxidant defenses over time [267].

#### 7.2.2. Tissue-Specific Redox Balance

The effects of radon exposure on redox balance vary across different tissues, depending on their intrinsic antioxidant capacity. Research investigating the redox state of multiple organs in mice exposed to radon for varying periods revealed distinct changes in antioxidant and lipid peroxide (LPO) levels. Notably, organs with lower baseline antioxidant capacity, such as the brain, pancreas, and stomach, exhibited altered correlations between SODs and other oxidative markers following radon exposure. These findings suggest that radon influences tissue-specific oxidative stress responses, with potential adaptive effects [15]. A 2026 mouse study further demonstrated marked organ-specific responses to combined thermal treatment and radon inhalation, with enhanced antioxidant function observed particularly in the brain and colon, variable responses in the kidney, decreased antioxidant indices in the lung, and limited or no changes in several other organs [268]. These findings further indicate that antioxidant responses to radon-containing interventions should not be generalized across tissues.

Radon exposure has also been linked to the modulation of uric acid metabolism. A study demonstrated that radon exposure (2000 Bq/m^3^ for 24 h) inhibited the elevation of serum uric acid levels in mice treated with potassium oxonate (PO), a compound that induces hyperuricemia. This effect was attributed to the suppression of xanthine oxidase activity in the liver and the activation of antioxidative functions in the liver and kidneys [14].

#### 7.2.3. Oxidative Stress Regulation in Osteoarthritis (OA)

Radon-rich hot spring therapy has been investigated for its potential to modulate oxidative stress and antioxidant capacity in OA patients. A study conducted at the Przerzeczyn-Zdrój spa resort examined the effects of a 21-day spa treatment on hemoglobin levels in 122 OA patients. A slight decrease in the mean hemoglobin level, from 14.1549 g% before treatment to 14.0008 g% after treatment, was observed. This reduction may be associated with free radical reactions induced by radon therapy [250].

In addition to hemoglobin level changes, radon hot springs have been shown to elevate SOD and catalase activities while suppressing lipid peroxidation and total cholesterol production, indicating enhanced antioxidant defense mechanisms [269]. Further studies assessing the antioxidant capacity and inflammatory markers in OA patients undergoing radon spa therapy reported an increase in total antioxidant status (TAS) and a significant reduction in prolactin levels post-treatment. These findings suggest that radon therapy may contribute to oxidative stress regulation and inflammation reduction in OA management [251]. Moreover, a study involving 35 patients with degenerative joint and disk disease found that radon water therapy led to an increase in TAS levels, while the control group exhibited a decrease. These results imply that low-dose ionizing radiation from radon exposure may enhance the integrated antioxidant system and contribute to oxidative stress regulation in OA patients [14].

### 7.3. Modulation of Immune Function

Radon therapy has been shown to modulate the immune system, with studies in both animal models and humans providing evidence of its effects on immune responses.

#### 7.3.1. Studies in Animal Models

Animal studies suggest that radon exposure can significantly influence immune function through various mechanisms. In experiments with BALB/c and SKG/Jcl mice, radon exposure at 2000 Bq/m^3^ for 2 or 4 weeks resulted in the activation of Th1, Th2 and Th17 immune responses, without disrupting the balance among these responses. Furthermore, radon inhalation led to reduced superoxide dismutase activity and increased catalase activity in the spleen, suggesting that oxidative stress may play a role in the activation of the immune system. Interestingly, in SKG/Jcl mice pre-treated with zymosan, which decreases Th1 and Th2 responses while activating Th17 responses, radon inhalation restored most cytokine levels related to Th1, Th2 and Th17 immune responses to normal levels, indicating that radon may have immunomodulatory effects [123].

Another study using K/BxN serum-induced arthritic mice showed that radon exposure in a controlled chamber significantly improved the clinical disease progression score. There was also a notable increase in peripheral blood B cells and IL-5 levels. However, no significant changes were observed in the antioxidative systems or cell death, suggesting that the beneficial effects were linked to immune system modulation rather than changes in oxidative stress or cell survival [14]. Additionally, a study involving male BALB/c mice exposed to 2000 Bq/m^3^ radon demonstrated that subsequent lipopolysaccharide (LPS) administration induced elevated levels of Th1- and Th17-related cytokines, and radon exposure suppressed these cytokine elevations, indicating an immunomodulatory role, even though it did not inhibit oxidative stress induced by LPS [14].

#### 7.3.2. Clinical Observations in Humans

Clinical studies have shown that radon therapy can have significant effects on immune function in humans. In a cohort of 103 patients with chronic musculoskeletal disorders, the serum levels of pro-inflammatory and anti-inflammatory cytokines were measured before and after therapy. The results indicated that TGFβ levels temporarily elevated at 6 weeks post-therapy, correlating with reduced pain sensitivity. Additionally, serum IL-18 levels were positively associated with decreased pain sensitivity, suggesting IL-18 as a potential marker for pain reduction following radon therapy [14].

Moreover, a 15-month low-dose radon and γ-radiation therapy regimen applied to a patient with rheumatoid arthritis (RA) resulted in near-complete recovery, with inflammation markers such as C-reactive protein (CRP) and matrix metalloproteinase-3 (MMP-3) returning to normal levels, indicating a long-term adaptive immune response [270]. In a separate study involving RA and osteoarthritis patients, low-dose radon therapy combined with hyperthermia (LDRnHT) led to a reduction in TNF-α and RANKL levels, suggesting a decrease in osteoclast activation. Notably, in RA patients, the increase in OPG levels resulted in a decreased RANKL/OPG ratio, which may contribute to reduced bone degradation. Furthermore, a significant decline in anti-CCP antibodies (ACPA) was observed, suggesting potential disease-modifying effects [14].

In addition to the aforementioned studies, further clinical research has indicated that radon therapy can positively influence bone metabolism and immune cell activity. A study involving 32 patients with MSD treated at a radon gas spa in Bad Steben, Germany, revealed changes in bone metabolism markers and adipokines. Specifically, serum levels of collagen fragments (CTX-I) decreased, suggesting reduced bone resorption due to decreased osteoclast activity. Moreover, increased regulatory T cells and decreased adiponectin levels were observed, indicating anti-inflammatory effects [14]. Another study showed that radon therapy augmented the mitogen response induced by concanavalin A, raising the proportion of CD4^+^ T cells, while reducing the proportion of CD8^+^ T cells. This suggests that radon therapy might influence helper T cell and suppressor T cell populations, modulating immune responses [269].

Finally, radon exposure in individuals with chronic painful degenerative musculoskeletal diseases has also been linked to significant long-term pain reduction and modulation of peripheral immune cells. This includes a reduction in the activation marker CD69 on T, B, and NK cells, and an increase in the percentage of HLA-DR^+^ T cells [14]. Additionally, a study of residents near radon-rich hot springs showed increased CD3^+^ and CD4^+^/CD8^−^ T cells in the radon-exposed group, suggesting that radon exposure could potentially alter immune functions [271].

## 8. Conclusions and Future Directions

Radon exposure is a well-established environmental health hazard and a confirmed cause of lung cancer, whereas its possible associations with other diseases remain under investigation. While much research has explored its carcinogenic and biological effects, several underlying mechanisms remain inadequately studied. Notably, the role of newer forms of cell death, such as ferroptosis (iron-dependent cell death) and cuproptosis (copper-dependent cell death), in radon-induced cellular damage remains largely unexplored [272,273]. These forms of cell death may play a significant role in the pathology of radon exposure and could provide novel insights into its detrimental effects. Understanding how these pathways intersect with radon-induced oxidative stress, DNA damage, and inflammatory responses could uncover new avenues for intervention and mitigation strategies in radon-exposed populations.

(1) In addition to research on the harmful effects of radon exposure, controlled low-dose radon exposure has also been investigated for potential therapeutic effects in selected conditions [14,229]. However, the precise biological mechanisms through which low-dose radon exposure exerts therapeutic effects remain incompletely understood. It is hypothesized that radon modulates oxidative stress, immune function, and cellular repair, but the specific molecular targets and pathways require further elucidation. Future research should determine whether and under what exposure conditions low-dose radon modifies antioxidant responses, inflammatory pathways, and immune function at the cellular and systemic levels [14,274]. Additionally, low-dose radon exposure has been linked to hormetic effects, where low radiation doses trigger adaptive responses that may protect against subsequent damage [275]. Investigating these adaptive mechanisms could provide deeper insights into the therapeutic potential of radon.
(2) The potential future clinical application of radon therapy requires further rigorous evaluation. Current studies have reported biological or symptomatic changes following low-dose radon exposure in some chronic inflammatory conditions, but the available evidence remains insufficient to establish therapeutic efficacy [14,270]. Identifying optimal dosing regimens, evaluating the safety and long-term effects of repeated exposure, and determining effective delivery methods are crucial steps in translating radon therapy into clinical practice. The traditional use of radon spas [276], where radon-rich mineral waters have been used for centuries for therapeutic purposes, further highlights the need to investigate the biological effects of radon-rich thermal waters. A better understanding of the biological effects and dosimetry of controlled radon exposure will be necessary before standardized therapeutic protocols can be considered. Any consideration of broader clinical implementation would require evidence from adequately powered controlled trials, standardized exposure and dosimetry protocols, long-term safety assessment, and appropriate regulatory oversight. However, clinical implementation requires strict regulation, long-term safety evaluations, and well-designed clinical trials to establish efficacy and minimize potential risks [277].
(3) Advancements in biomedical technology will play a critical role in uncovering the precise mechanisms underlying radon’s effects. Single-cell sequencing techniques offer powerful tools for investigating radon-induced cellular responses with unprecedented resolution. Single-cell RNA sequencing (scRNA-Seq) enables the study of transcriptional heterogeneity among individual cells [278], revealing distinct cellular responses to radon exposure. scDNA-Seq can identify genomic mutations and copy number variations resulting from radon-induced damage [279]. Additionally, single-cell epigenomics, including DNA methylation profiling and chromatin accessibility assays (such as ATAC-seq), can provide insights into how radon affects gene regulation at the epigenetic level [280,281]. Multi-omics approaches integrating transcriptomics, genomics, proteomics, and metabolomics at the single-cell level will be particularly valuable for dissecting the complex biological effects of radon exposure [282]. These technologies could help distinguish beneficial adaptive responses from detrimental damage, thereby informing both risk assessment and therapeutic applications of radon exposure.
(4) Post-translational modifications (PTMs) induced by ionizing radiation represent another crucial area of molecular response. It has been reported that ionizing radiation can induce PTMs like phosphorylation, acetylation, ubiquitination, and S-nitrosylation, which influence cellular stress responses, DNA repair pathways, and cell survival, which play key roles in processes like DNA damage response, cell cycle regulation, and apoptosis, providing potential targets for developing new radiation therapy strategies [283]. For example, ionizing radiation can decrease protein S-nitrosylation levels, exacerbating ROS generation and enhancing radiosensitivity [284]. Future studies should explore whether radon exposure triggers similar PTMs, how these modifications affect cellular function, and whether targeting PTM-related pathways could mitigate radon-induced damage or enhance its therapeutic effects.

Several limitations of this review should be acknowledged. As a comprehensive narrative review, the literature was not selected according to a formal systematic-review protocol, and no standardized quality or risk-of-bias assessment was applied to the included studies. In addition, the strength of evidence varies substantially across topics; in particular, some therapeutic applications are supported mainly by case reports, small observational studies, or non-randomized clinical investigations. Consequently, conclusions in these areas should be interpreted cautiously, and future systematic reviews and well-designed controlled studies are needed to strengthen the evidence base. Moreover, studies of radon therapy vary substantially in exposure route, radon concentration, treatment duration, concomitant interventions, study design, and outcome assessment. These sources of heterogeneity limit direct comparison across studies, and no quantitative estimate of an overall therapeutic effect should be inferred from this review.

Although biological and symptomatic effects have been reported following controlled radon exposure, substantial uncertainty remains regarding clinical efficacy, optimal exposure conditions, and long-term safety. Interdisciplinary collaboration involving radiobiologists, immunologists, and clinicians will be crucial in addressing gaps in our understanding of radon’s biological effects and therapeutic applications. Future research should clarify the underlying mechanisms of radon exposure and determine whether reported therapeutic effects are reproducible and clinically meaningful across specific indications. Any transition from experimental or spa-based applications to broader clinical use should be guided by rigorous evidence regarding efficacy, dosimetry, long-term safety, and benefit–risk balance. For indications currently supported mainly by preclinical studies or case reports, including most oncological applications, therapeutic efficacy remains to be established.

## Figures and Tables

**Table 1:** Clinical and preclinical studies of radon therapy and its therapeutic applications.

First Author & Year of Publication	Location	Sample Size & Indications	Trial Design	Conclusions	Reference
Becker, K., 2003	Germany and Austria	Not included	Review/summary of experience	Radon therapy may provide symptomatic benefits in selected rheumatic conditions, but the balance of benefits and risks remains uncertain.	[210]
Herold, M. & Lind-Albrecht, 2008	Gastein Valley, Austria	Hundreds; AS	Review of clinical experience and controlled trials	Radon-containing spa therapy may provide sustained symptomatic improvement in patients with ankylosing spondylitis.	[211]
Shehata, M., 2006	Badgastein, Austria	83; AS	Prospective clinical study with control groups	Combined spa-exercise therapy was associated with increased circulating TGF-β1 levels; the independent effect of radon cannot be determined.	[212]
van der Zee-Neuen, A., 2022	Austrian Gastein Valley	AS; registry-based cohort	Prospective registry data analysis	Spa therapy including low-dose radon was associated with sustained improvements in quality of life for up to 6–9 months.	[213]
Winklmayr, M., 2015	Austria	Participants at risk of osteoporosis	Randomized placebo-controlled intervention study	Combined radon balneotherapy and exercise showed changes in bone-related markers, but the independent effect of radon was limited.	[214]
Polshchakova, T., 2022	Ukraine	40; osteochondrosis with radicular syndrome	Randomized controlled clinical trial	Radon baths were associated with reduced pain and improved spinal mobility in patients with osteochondrosis.	[215]
Kuciel-Lewandowska, J., 2015	Przerzeczyn-Zdrój, Poland	120; degenerative joint and disk disease	Observational comparative study	Comprehensive spa therapy was associated with greater pain reduction; the independent effect of radon cannot be determined.	[216]
Razumov, A. N., 2015	Russia	75; knee osteoarthritis	Comparative clinical study	Combined radon baths and extracorporeal shock-wave therapy were associated with reduced pain and improved joint function; radon-specific effects cannot be isolated.	[217]
Vakulenko, O. Y., 2017	Russia	Not included; OA	Narrative literature review	Cryotherapy and radonotherapy may support OA rehabilitation, but their independent effects cannot be determined from combined treatment evidence.	[218]
Ilyinskikh, N. N., 2018	Belokurikha, Russia	121; gonosteoarthritis associated with Lyme borreliosis or post-traumatic OA	Comparative clinical study	Radon bath therapy was associated with reduced pain and fewer synovial cells with chromosome 7 trisomy.	[219]
Gaisberger, M., 2021	Bad Gastein, Austria	25; knee OA	Randomized controlled open pilot study	Multimodal spa treatment with or without radon was associated with sustained pain reduction; radon-specific effects remained limited.	[220]
Annegret, F. & Thomas, 2013	Germany and Austria	681; chronic back pain, OA, RA, and/or AS	Randomized blinded multicenter controlled trial	Radon spa therapy was associated with longer-term pain reduction in patients with chronic rheumatic diseases.	[221]
Stanciak, J., 2020	Czech Republic	50; rheumatoid arthritis	Pilot observational study	Spa radon therapy was associated with improved quality-of-life measures in patients with rheumatoid arthritis.	[222]
Franke, A., 2000	Germany	134; rheumatoid arthritis	Randomized, sham-controlled clinical trial	Radon spa therapy was associated with longer-term symptomatic benefit in rheumatoid arthritis.	[223]
Van der Zee-Neuen, A., 2023	Austrian Gastein Valley	561; RMD	Longitudinal registry data analysis	Multimodal spa therapy was associated with sustained pain reduction; the specific contribution of radon cannot be isolated.	[224]
Falkenbach, A., 2005	Not applicable	338 participants across included trials; rheumatic diseases	Systematic review and meta-analysis of controlled clinical trials	Controlled trials suggested longer-term pain reduction after radon therapy in rheumatic diseases.	[14]
Efimenko, 2014	Russia	100; recurrent erosive and ulcerative lesions of the esophagus, stomach, and duodenum	Clinical comparative study	Spa-based rehabilitation was associated with clinical improvement, but the contribution of individual treatment components cannot be isolated.	[225]
Etani, R., 2017	Not included	Mice; ethanol-induced gastric mucosal injury	Preclinical animal experiment	Hot spring water drinking and radon inhalation reduced ethanol-induced gastric mucosal injury in mice.	[226]
Kataoka, T., 2023	Misasa, Japan	5250; general health status	Cross-sectional observational study	Radon hot spring bathing was associated with better self-rated health and lower reported hypertension and gastroenteritis.	[227]
Kataoka, T., 2014	Not included	Gerbils; transient global cerebral ischemia	Preclinical animal experiment	Radon inhalation increased antioxidant activity and reduced ischemic neuronal injury in gerbils.	[228]
Kataoka, T., 2016	Japan	5–6 mice; neuropathic pain	Preclinical animal experiment	Radon inhalation enhanced pregabalin-associated pain relief in mice, possibly through antioxidative mechanisms.	[229]
Kanzaki, N., 2022	Japan	Mice; radon exposure	Preclinical animal experiment	Radon inhalation altered sulfur-related metabolites in mouse brain under different exposure conditions.	[230]
Dondoladze, K., 2021	Georgia	Rats; oxidative stress and anxiety	Preclinical animal experiment	Radon-containing balneotherapy enhanced antioxidant responses and showed anxiolytic effects in rats.	[231]
Marullo, T., 2000	Merano Spa, Italy	27; chronic rhinosinusitis	Controlled clinical study	Radon-enriched inhalation was associated with improved nasal airflow and mucociliary clearance.	[232]
Passali, D., 2012	Merano Therme, Italy	33; chronic sinonasal inflammation	Prospective pre-post clinical study	Radioactive thermal-water inhalation was associated with improved mucociliary function and nasal cytology.	[233]
Passali, D., 2016	Merano SPA, Italy	90; allergic rhinitis	Prospective comparative clinical study	Radioactive thermal-water inhalation was associated with clinical and cytological improvement comparable to mometasone nasal spray.	[234]
Passali, D., 2017	Not applicable	Four prospective studies; rhinitis, rhinosinusitis, and asthma	Review of clinical studies	Radon-enriched inhalation therapy was associated with improved objective respiratory-function measures.	[209]
Kojima, 2018	Japan	4 cancer cases/review-based discussion	Review with case reports	Radon and other α-emitting radionuclides were discussed as potential therapeutic approaches, but clinical evidence remained limited.	[235]
Takahashi, 2006	Japan	Mice; atopic dermatitis and melanoma metastasis	Preclinical animal experiment	Low-level radon exposure suppressed atopic dermatitis and tumor metastasis in mouse models.	[236]
Kojima, 2019	Japan	4 patients; colon, uterine, lung, and hepatocellular cancers	Case series	Radon inhalation was associated with clinical responses in four cancer cases, but efficacy cannot be established from case reports.	[237]
Nikolaishvili, 2018	Tskhaltubo, Georgia	150; 120 periodontitis patients and 30 healthy controls	Case–control clinical study	Tskhaltubo radon-containing mineral-water inhalation and rinsing were associated with improved periodontal inflammatory indicators.	[238]
Omiadze, 2020	Tskhaltubo, Georgia	132; periodontitis	Comparative clinical study	Radon-containing water inhalation and rinsing were associated with reduced oral LDH levels and improved biochemical indicators.	[239]
Dolidze, 2021	Tskhaltubo, Georgia	Periodontitis patients	Combined clinical intervention study	Combined photodynamic therapy and Tskhaltubo radon water were associated with improvement in periodontal inflammation; the independent effect of radon cannot be determined.	[240]
Akhkubekova, N. K., 2018	Pyatigorsk, Russia	89; endometriosis genitalis externa	Randomized comparative clinical study	Combined balneotherapy with radon and GnRH-agonist therapy was associated with improved outcomes; the independent effect of radon cannot be determined.	[241]
Shchegolikhina, L. V., 2019	Russia	348; stages I–III endometriosis	Clinical treatment study/staged rehabilitation approach	A staged multimodal rehabilitation approach was associated with improved outcomes in women with endometriosis.	[242]
Deloch, L., 2022	Germany	BALB/c and SKG/Jcl mice; rheumatoid arthritis model	Preclinical animal experiment	Radon exposure improved clinical arthritis scores and altered peripheral immune-cell profiles in mice.	[243]
Kataoka, T., 2022	Japan	Mice; rheumatoid arthritis model	Preclinical animal experiment	Radon inhalation modulated cytokine responses in normal and rheumatoid-arthritis mouse models.	[123]
Kullmann, M., 2018	Germany	103; chronic painful musculoskeletal disorders	Prospective exploratory clinical study	Radon spa therapy was associated with transient TGFβ elevation and lower pain sensitivity; causality cannot be established from this design.	[244]
Cucu, A., 2017	Bad Steben, Germany	32; musculoskeletal disorders	Prospective exploratory clinical study	Serial low-dose radon spa therapy was associated with reduced serum CTX-I levels, suggesting altered bone resorption.	[245]
Rühle, P. F., 2017	Germany	100; chronic painful degenerative musculoskeletal diseases	Prospective exploratory clinical study	Radon spa therapy was associated with modulation of peripheral immune-cell activation markers.	[246]
Lange, U., 2016	Bad Gastein, Austria	49; 25 RA and 24 OA patients	Prospective comparative clinical study	Serial radon-hyperthermia exposure was associated with favorable changes in cytokines related to bone metabolism.	[247]
Kataoka, T., 2021	Japan	Mice; oxidative DNA damage	Preclinical animal experiment	Radon inhalation increased Mn-SOD activity and was associated with reduced oxidative DNA damage in mouse organs.	[248]
Etani, R., 2016	Japan	Mice; hyperuricemia	Preclinical animal experiment	Radon inhalation suppressed hyperuricemia in mice through activation of antioxidative functions.	[249]
Kuciel-Lewandowska, J., 2020	Przerzeczyn-Zdrój, Poland	122; OA or disk herniation	Prospective pre-post clinical study	Comprehensive radon-sulfur spa therapy was associated with a small decrease in hemoglobin; radon-specific effects cannot be isolated.	[250]
Kuciel-Lewandowska, J., 2020	Poland	50; OA (35 treatment, 15 control)	Controlled prospective clinical study	Complex spa treatment with radon water was associated with changes in TAS; the independent effect of radon cannot be determined.	[251]
Kuciel-Lewandowska, J., 2018	Poland	50; OA (35 radon-treated, 15 control)	Controlled pilot clinical study	Radon water therapy was associated with increased TAS in the treatment group, but larger studies are required.	[252]

## Data Availability

No new data were created or analyzed in this study. Data sharing is not applicable to this article.

## Abbreviations

Abbreviation	Full name
AD	Alzheimer’s disease
ALL	Acute lymphoblastic leukemia
AMD	Arithmetic mean diameter
AML	Acute myeloid leukemia
AS	Ankylosing spondylitis
ATM	Ataxia-telangiectasia mutated
BCC	Basal cell carcinoma
BE	Bystander effect
cf mtDNA	Circulating free mitochondrial DNA
circRNAs	Circular RNAs
CLL	Chronic lymphocytic leukemia
COPD	Chronic obstructive pulmonary disease
CRP	C-reactive protein
DaRT	Diffusing α-emitter radiation therapy
DDR	DNA damage response
DSBs	Double-strand breaks
EDE	Enhanced deposition effect
EGFR	Epidermal growth factor receptor
EMT	Epithelial–mesenchymal transition
ESTs	Expressed sequence tags
FASN	Fatty acid synthase
HPRT	Hypoxanthine guanine phosphoribosyl transferase
IARC	International Agency for Research on Cancer
IDRE	Inverse dose-rate effect
LDH	Lactate dehydrogenase
LDL	Low-density lipoproteins
LET	Linear energy transfer
lncRNAs	Long non-coding RNAs
LPS	Lipopolysaccharide
mFISH	Multicolor fluorescence in situ hybridization
miRNAs	MicroRNAs
MMP	Mitochondrial membrane potential
MMPs	Matrix metalloproteinases
MN	Micronucleus
mRNAs	Messenger RNAs
MS	Multiple sclerosis
MSD	Musculoskeletal disease
mtDNA	Mitochondrial DNA
ncRNAs	Non-coding RNAs
NHEJ	Non-homologous end joining
NSCLC	Non-small-cell lung cancer
NTDs	Nuclear track detectors
OA	Osteoarthritis
PD	Parkinson’s disease
PSA	Prostate-specific antigen
PTK	Protein tyrosine kinase
PTMs	Post-translational modifications
RA	Rheumatoid arthritis
RAR	Radiation adaptation response
RBE	Relative biological effectiveness
RIGI	Radiation-induced genomic instability
Rn	Radon
ROS	Reactive oxygen species
SCC	Squamous cell carcinoma
SCFAs	Short-chain fatty acids
SCLC	Small-cell lung cancer
SNPs	Single-nucleotide polymorphisms
SOD	Superoxide dismutase
TAS	Total antioxidant status
TK	Thymidine kinase
UV	Ultraviolet
VEGF	Vascular endothelial growth factor
WHO	World Health Organization
WLM	Working level months
